# Cripto‐1 acts as a molecular bridge linking nodal to ALK4 via distinct structural domains

**DOI:** 10.1002/pro.70034

**Published:** 2025-01-22

**Authors:** Kit‐Yee Chu, Amberly N. Crawford, Bradon S. Krah, Vijayalakshmi Thamilselvan, Anjali Malik, Nina A. Aitas, Erik Martinez‐Hackert

**Affiliations:** ^1^ Department of Biochemistry and Molecular Biology Michigan State University East Lansing Michigan USA; ^2^ Department of Structural Biology Van Andel Institute Grand Rapids Michigan USA

**Keywords:** ALK4, AlphaFold, co‐receptor, Cripto‐1, EGF‐CFC proteins, molecular modeling, nodal, protein–protein interaction, signaling transduction, transforming growth factor beta (TGF‐*β*)

## Abstract

The TGF‐*β* family ligand Nodal is an essential regulator of embryonic development, orchestrating key processes such as germ layer specification and body axis formation through activation of SMAD2/3‐mediated signaling. Significantly, this activation requires the co‐receptor Cripto‐1. However, despite their essential roles in embryogenesis, the molecular mechanism through which Cripto‐1 enables Nodal to activate the SMAD2/3 pathway has remained elusive. Intriguingly, Cripto‐1 also has been shown to antagonize other TGF‐*β* family ligands, raising questions about its diverse functions. To clarify how Cripto‐1 modulates TGF‐*β* signaling, we integrated AlphaFold3 modeling, surface plasmon resonance (SPR)‐based protein–protein interaction analysis, domain‐specific anti‐Cripto‐1 antibodies, and functional studies in NTERA‐2 cells. In contrast to canonical TGF‐*β* signaling, where ligands bridge type I and type II receptors for activation, Nodal, bound to the type II receptor, utilizes Cripto‐1 to recruit the type I receptor ALK4, forming a unique ternary complex for SMAD2/3 activation. Our molecular characterization of Cripto‐1‐mediated Nodal signaling clarifies the unique role of this enigmatic co‐receptor and advances our understanding of signaling regulation within the TGF‐*β* family. These insights have potential implications for both developmental biology and cancer research.

## 1 INTRODUCTION

Embryonic development is a tightly regulated process governed by a complex network of signaling pathways (Sonnen & Janda, 2021). These pathways enable communication between cells, orchestrating developmental outcomes by directing cell fate decisions and the formation of tissues (Perrimon et al., 2012). Central to this regulatory signaling network is the transforming growth factor‐*β* (TGF‐*β*) family (Wu & Hill, 2009; Zinski et al., 2018). This family comprises structurally homologous ligands, including TGF‐*β*s, bone morphogenetic proteins (BMPs), Activins, and Nodal (Hinck et al., 2016). These ligands bind to cell‐surface receptors and co‐receptors, triggering SMAD transcription factor‐mediated signaling cascades that directly regulate the expression of genes essential for cell fate determination and tissue patterning (Shi & Massague, 2003; Wu & Hill, 2009).

Among members of the TGF‐*β* family, the ligand Nodal and the co‐receptor Cripto‐1 are fundamental to embryogenesis, guiding germ layer specification and body axis formation (Adachi et al., 1999; Ang & Constam, 2004; Schier, 2003). Like other TGF‐*β* family ligands, Nodal signals by forming a complex with both type I and type II TGF‐*β* family receptors (Aykul et al., 2015; Gu et al., 1998; Heldin & Moustakas, 2016; Reissmann et al., 2001). However, Nodal signaling uniquely depends on the glycosylphosphatidylinositol (GPI)‐anchored co‐receptor Cripto‐1, the founding member of the epidermal growth factor (EGF)‐Cripto‐1/FRL‐1/Cryptic (CFC) co‐receptor family (Bianco et al., 2002; Gritsman et al., 1999; Yan et al., 1999; Yan et al., 2002; Yeo & Whitman, 2001). Substantial evidence supports this interaction. For instance, zebrafish mutants of Nodal and Cripto‐1 exhibit phenocopy (Gritsman et al., 1999) and cell‐based assays have indicated that Nodal requires Cripto‐1 to induce downstream signaling (Schiffer et al., 2001; Yeo & Whitman, 2001). In addition, biochemical assays confirm that Cripto‐1 and Nodal interact. Their direct binding was demonstrated using surface plasmon resonance (SPR) and co‐immunoprecipitation (co‐IP) (Aykul et al., 2015; Yan et al., 2002; Yeo & Whitman, 2001). This evidence, combined with the co‐IP of Cripto‐1 with TGF‐*β* family receptors (Gray et al., 2003; Yeo & Whitman, 2001), supports a model in which Cripto‐1 interacts with Nodal and type I or type II Nodal receptors to facilitate the formation of an active Nodal signaling complex.

However, despite evidence suggesting that Cripto‐1 plays a crucial role in Nodal signaling, the molecular basis underlying their interaction remains unclear. Moreover, some studies indicate that Nodal signaling can occur independently of Cripto‐1, as murine Cripto‐1 and Nodal mutants do not phenocopy exactly (Liguori et al., 2008). This discrepancy implies that Cripto‐1 may have Nodal‐independent functions or that Nodal may also signal independently of Cripto‐1 (Yeo & Whitman, 2001). Furthermore, Cripto‐1 has been implicated in regulating the activity of other TGF‐*β* family members, suggesting a broader role in modulating the TGF‐*β* signaling network. In fact, several studies have reported that Cripto‐1 can inhibit the signaling of other TGF‐*β* family members, including Activins, TGF‐*β*1, and BMP‐4 (Adkins et al., 2003; Aykul et al., 2017; Gray et al., 2003, 2006; Kelber et al., 2008). These findings suggest that Cripto‐1 could have dual, ligand‐dependent roles: it may serve as a co‐receptor to enable Nodal signaling and act as an antagonist to suppress the signaling of other TGF‐*β* family members. These multifaceted roles have made elucidating the mechanisms of Cripto‐1 action challenging.

While we sought to determine the molecular basis of Cripto‐1 interaction through experimental structures of Cripto‐1 or its complexes, this effort proved to be an intractable challenge due to the inherent flexibility of Cripto‐1 and the difficulties associated with expressing and purifying its ligand, Nodal. However, recent advances in protein structure prediction, particularly AlphaFold3 (Abramson et al., 2024), have provided powerful new tools to investigate the structure and function of challenging proteins like Cripto‐1. Using AlphaFold3, we generated structural models of Cripto‐1 complexes that allowed us to develop a molecular hypothesis of Cripto‐1 action. In this model, Cripto‐1 functions as an essential adaptor protein, bringing the type I receptor ALK4 into proximity with the Nodal–ACVR2B complex. This interaction forms a complex resembling a canonical TGF‐*β* family signaling complex, which typically includes two type I and two type II receptors. We employed a combination of approaches, including Cripto‐1 domain constructs (Aykul et al., 2017), domain‐specific monoclonal antibodies and protein–protein interaction assays, to validate these models. To further explore the functional consequences of these interactions, we used NTERA‐2 cells, which endogenously express Cripto‐1, and investigated the effects of Cripto‐1 and its domain‐specific antibodies on Nodal signaling.

By integrating findings from these diverse approaches, we have constructed a biochemically consistent model. This model explains how Cripto‐1 interacts with Nodal and the type I TGF‐*β* family receptor ALK4, offering a potential framework for understanding its role as a co‐receptor that is required for Nodal signaling. These insights are particularly significant during embryonic development and in cancers where Cripto‐1 has a critical role.

## 2 RESULTS

### 2.1 AlphaFold3 insights into Cripto‐1 complexes

**FIGURE 1:**
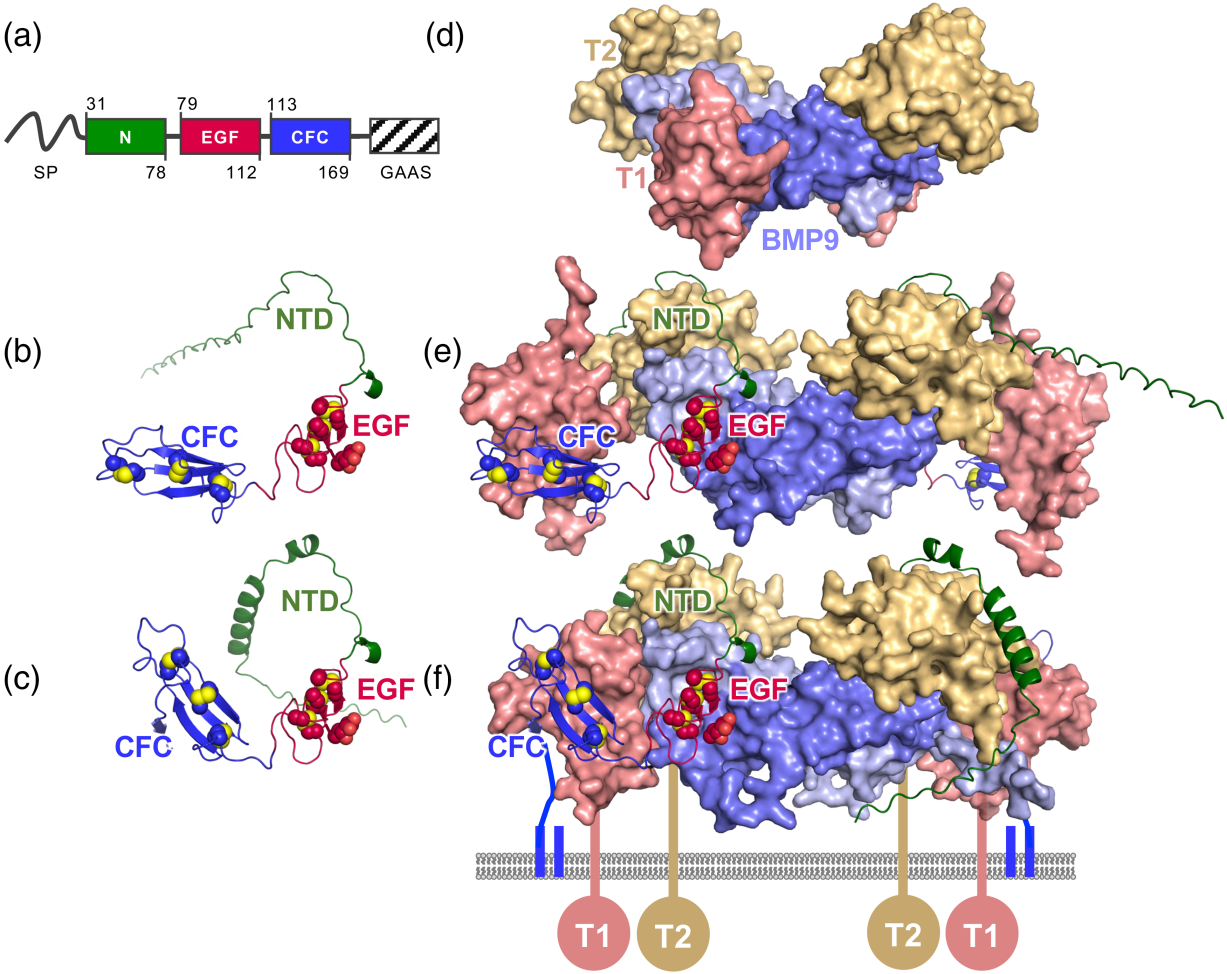
AlphaFold3‐predicted structures of human and Zebrafish Cripto‐1 and their interactions in the Nodal signaling complex. (a) Schematic of Cripto‐1 domain architecture, highlighting the N‐terminal (N) domain (green), EGF‐like domain (red), and CFC domain (blue). The secretion signal peptide is represented as a black line, the GPI‐anchor signal peptide as a striped box, and residue numbers corresponding to human Cripot‐1 are indicated. (b) AlphaFold3‐predicted model of human Cripto‐1 (UniProt ID: P13689) with domains colored as in (a). Residues 31–161, N‐linked glycan at Asn79 and O‐linked Fucose at Threonine 88 (T88) were included in the modeling. (c) AlphaFold3‐predicted model of zebrafish Cripto (Oep) (UniProt ID: Q90X11) with domains colored as in (a). Residues 21–168 and O‐linked Fucose at T89 were included in the modeling. (d) Crystal structure of the BMP9–ALK1–ACVR2B complex (PDB ID: 3Q4U) representing a canonical TGF‐*β* signaling complex. The ligand BMP9 is shown in light blue, the type I receptor ALK1 in salmon, and the type II receptor ACVR2B in gold. (e) AlphaFold3‐predicted model of the human Cripto‐1–Nodal–ALK4–ACVR2B signaling complex, showing Cripto‐1 bridging Nodal to ALK4. Nodal, ALK4, and ACVR2B are represented as molecular surfaces, shown in light blue, salmon, and gold, respectively. Cripto‐1 is represented as a ribbon diagram with domains colored as in (a). The following proteins and residues were used in the modeling: Cripto‐1 (UniProt ID: P13689) residues 31–161, N‐linked glycan at Asn79 and O‐linked Fucose at T88; ACVR2B (UniProt ID: Q13705) residues 19–119; ACVR1B (UniProt ID: P36896) residues 24–115; and Nodal (UniProt ID: Q96S42) residues 238–347. (f) AlphaFold3‐predicted model of the zebrafish Cripto‐1–Nodal–ALK4–ACVR2B homolog complex. The coloring scheme is the same as in (e), with the addition of transmembrane regions (colored lines), receptor kinase domains (colored circles), and the Cripto‐1 GPI anchor (blue double line). Nodal, ALK4, and ACVR2B are represented as molecular surfaces, and Cripto‐1 (Oep) is represented as a ribbon diagram. The following proteins and residues were used in the modeling: Oep (UniProt ID: Q90X11) residues 21–168 and O‐linked Fucose at T89; ACVR2B (UniProt ID: Q9YGU4) residues 23–121; ACVR1B (UniProt ID: P79689) residues 32–112; and Squint (UniProt ID: O13144) residues 263–392.

Cripto‐1 is a modular protein consisting of three distinct domains: an N‐terminal domain (N‐domain) with low sequence homology, a central EGF‐like domain (E‐domain), and a C‐terminal Cripto‐FRL1‐Cryptic (CFC) domain (C‐domain), which is the defining structural feature of the family (Figure 1a). The CFC domain is followed by a Glycosylphosphatidylinositol Anchor Attachment Signal sequence, which is cleaved during posttranslational modification and results in the attachment of Cripto‐1 to the cell membrane via a GPI anchor.

To gain insights into the molecular basis underlying Cripto‐1 function, we generated structural models of human Cripto‐1 and its zebrafish homolog, one‐eyed pinhead, using AlphaFold3 (Figure 1b, c). AlphaFold3 predicted the EGF‐like and CFC domains with high confidence, as reflected by high pLDDT and low PAE scores for these regions. As expected, the EGF‐like domain adopts a characteristic β‐hairpin fold stabilized by three disulfide bonds (Wouters et al., 2005), while the CFC domain adopts a compact structure characterized by three antiparallel *β*‐strands that are stabilized by three unique disulfide bonds (Calvanese et al., 2006). The structural conservation of these domains between human and zebrafish Cripto‐1 further supports the accuracy of the AlphaFold3 predictions (Figure S1). In contrast, the N‐terminal domain and the linker region between the CFC domain and the GPI anchor exhibited low sequence homology across species. This low homology resulted in reduced prediction accuracy, limiting our ability to draw strong conclusions about the roles of these regions.

To understand how Cripto‐1 functions as a co‐receptor in Nodal signaling, we modeled Nodal–Cripto‐1–receptor complexes. We then performed structural comparisons with known TGF‐*β* family receptor complexes to identify conserved mechanisms of receptor assembly. Specifically, we referenced the experimentally determined BMP9/ALK1/ACVR2B structure, which serves as a paradigm for canonical Activin/BMP receptor complexes (Figure 1d) (Townson et al., 2012). Active TGF‐*β* family signaling complexes typically comprise a dimeric ligand (BMP9), two type I receptors (ALK1), and two type II receptors (ACVR2B). This configuration brings all receptors into proximity within the signaling assembly, enabling type II receptors to phosphorylate type I receptors, thereby activating them and initiating a subsequent cascade of downstream signaling.

To obtain structural models of the human and zebrafish Cripto‐1–Nodal–ALK4–ACVR2B complexes, we utilized AlphaFold3 (Figure 1e,f for humans and zebrafish, respectively). Superposition of the human and zebrafish models revealed a high degree of structural conservation, increasing our confidence in their accuracy. Notably, the linker region between the Cripto‐1 EGF and CFC domains exhibited flexibility, resulting in variations in the orientation of the CFC domain relative to ALK4 (Figure S1A). Despite this variability, the specific molecular interactions between the different Cripto‐1 domains and their binding partners were conserved. This strong structural conservation between the human and zebrafish complexes, along with favorable pLDDT scores (generally above 90) and low predicted aligned error (PAE) scores (Figure S1B, C), supports the reliability of the AlphaFold3 models for predicting the structure of the Cripto‐1–Nodal–ALK4–ACVR2B complex.

**FIGURE 2:**
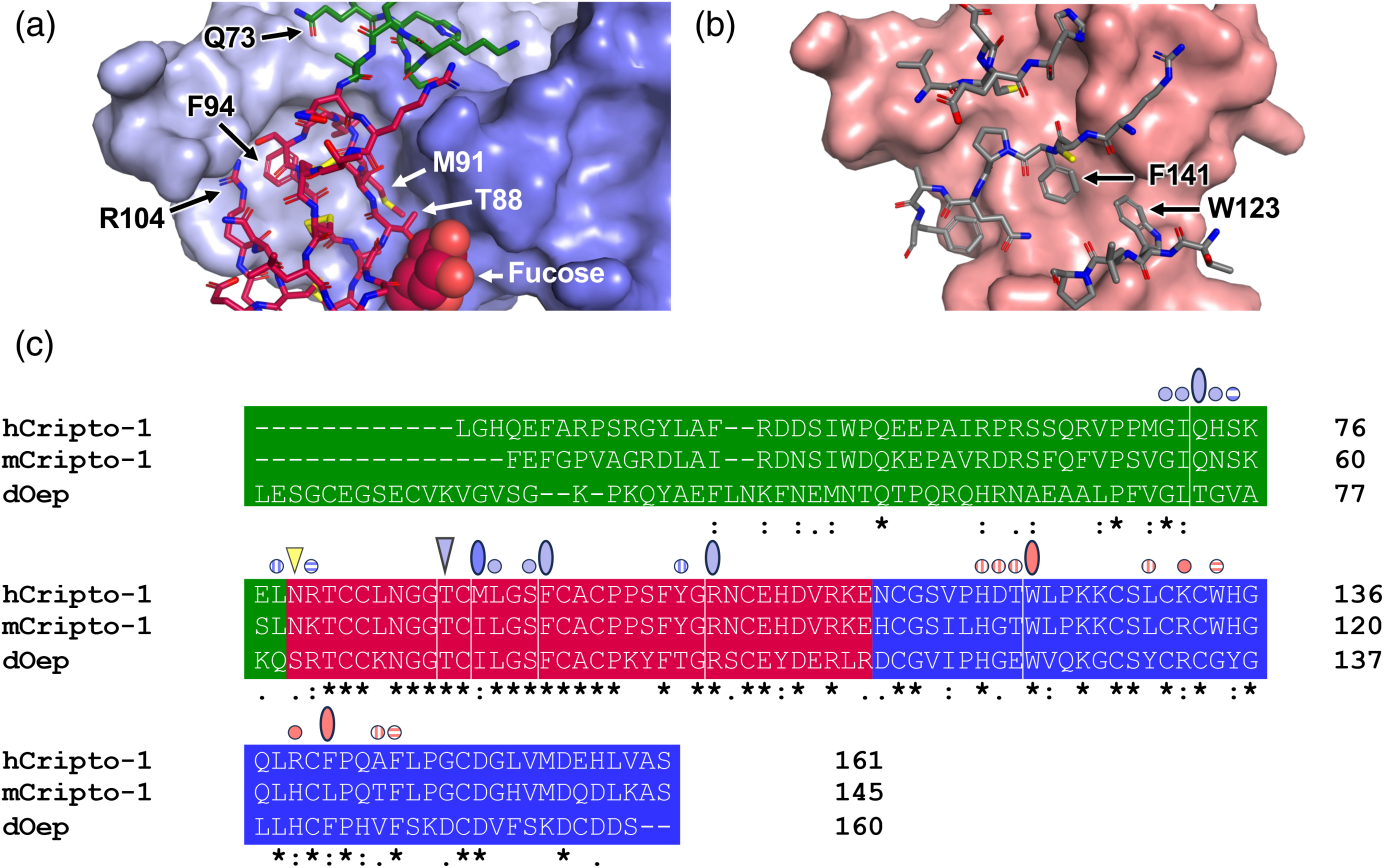
Close‐up views of Cripto‐1 binding interfaces with Nodal and ALK4, and analysis of interacting residues. (a) Zoomed‐in view of the interface between the Cripto‐1 EGF‐like domain (stick model) and Nodal (surface) from the AlphaFold3‐predicted model of the human Cripto‐1–Nodal–ALK4– ACVR2B signaling complex (Figure 1e). The fucosylated T88 of Cripto‐1, with the fucose moiety shown as spheres, is shown contacting Nodal at the canonical type I receptor binding site, typically the type I receptor binding site in other TGF‐*β* ligand‐receptor complexes. (b) Zoomed‐in view of the interface between the Cripto‐1 CFC domain (stick model) and the type I receptor ALK4 (surface) from the AlphaFold3‐predicted model of the human Cripto‐1–Nodal–ALK4–ACVR2B signaling complex (Figure 1e). The W23 residue of Cripto‐1 is found at the interface. The CFC domain binds ALK4 at the canonical ligand binding site on the type I receptor, typically the ligand binding site in other TGF‐*β* ligand‐receptor complexes. (c) Alignment of human, mouse, and zebrafish Cripto‐1 sequences used in AlphaFold3 modeling. Domains are highlighted as in Figure 1: The N‐terminal (N) domain (green), EGF‐like domain (red), and CFC domain (blue). The N‐linked glycan is noted by a yellow triangle, and the fucosylated threonine is highlighted by a blue triangle. Contacting residues identified with FoldScript are shown, where full circles represent residues identified in all 5 human and 5 zebrafish Alphafold3 models. Half‐filled circles represent residues identified in either human (vertical) or zebrafish (horizontal) models. Ovals represent residues shown in panels (a) or (b). Light blue circles or ovals represent Cripto‐1 residues interacting with one Nodal protomer (chain A). Dark blue circles or ovals represent residues interacting with the second Nodal protomer (chain B). The salmon‐colored circles/ovals represent Cripto‐1 residues interacting with ALK4.

AlphaFold3 predictions reveal distinct roles for the Cripto‐1 EGF‐like domain, CFC domain, and parts of the N‐domain within the putative signaling complex. The EGF‐like domain binds Nodal at a site typically occupied by type I receptors in ligand–receptor complexes, with approximately 10 residues at the C‐terminal end of the N‐domain predicted to provide additional contacts. By contrast, the CFC domain interacts with the canonical ligand‐binding surface of ALK4. Notably, the predicted binding interfaces for Nodal and ALK4 align with residues previously identified through mutagenesis studies as critical for these interactions (Schiffer et al., 2001; Shi et al., 2007; Yeo & Whitman, 2001). Specifically, these residues include the fucosylated threonine at position 88 (T88) within the EGF‐like domain (Figure 2a), whose mutation disrupts Nodal signaling (Schiffer et al., 2001; Shi et al., 2007), and the tryptophan at position 123 (W123) within the CFC domain (Figure 2b), whose mutation disrupts Cripto‐1–ALK4 co‐IP (Yeo & Whitman, 2001). The predicted contacts involving these residues are well conserved across all AlphaFold3 models and between the human and zebrafish complexes (Figures 2c and S2), further supporting these predicted structures. Furthermore, consistent with existing experimental structures, AlphaFold3 predicted a direct interaction between Nodal and the type II receptor ACVR2B at the conserved “knuckle” epitope region (Kirsch et al., 2000). Collectively, these predictions suggest that Cripto‐1 functions as a bridge, linking Nodal to ALK4 and facilitating the assembly of a unique signaling complex that incorporates both type I and type II receptors.

### 2.2 Domain deletion constructs confirm Nodal binding to EGF domain

**FIGURE 3:**
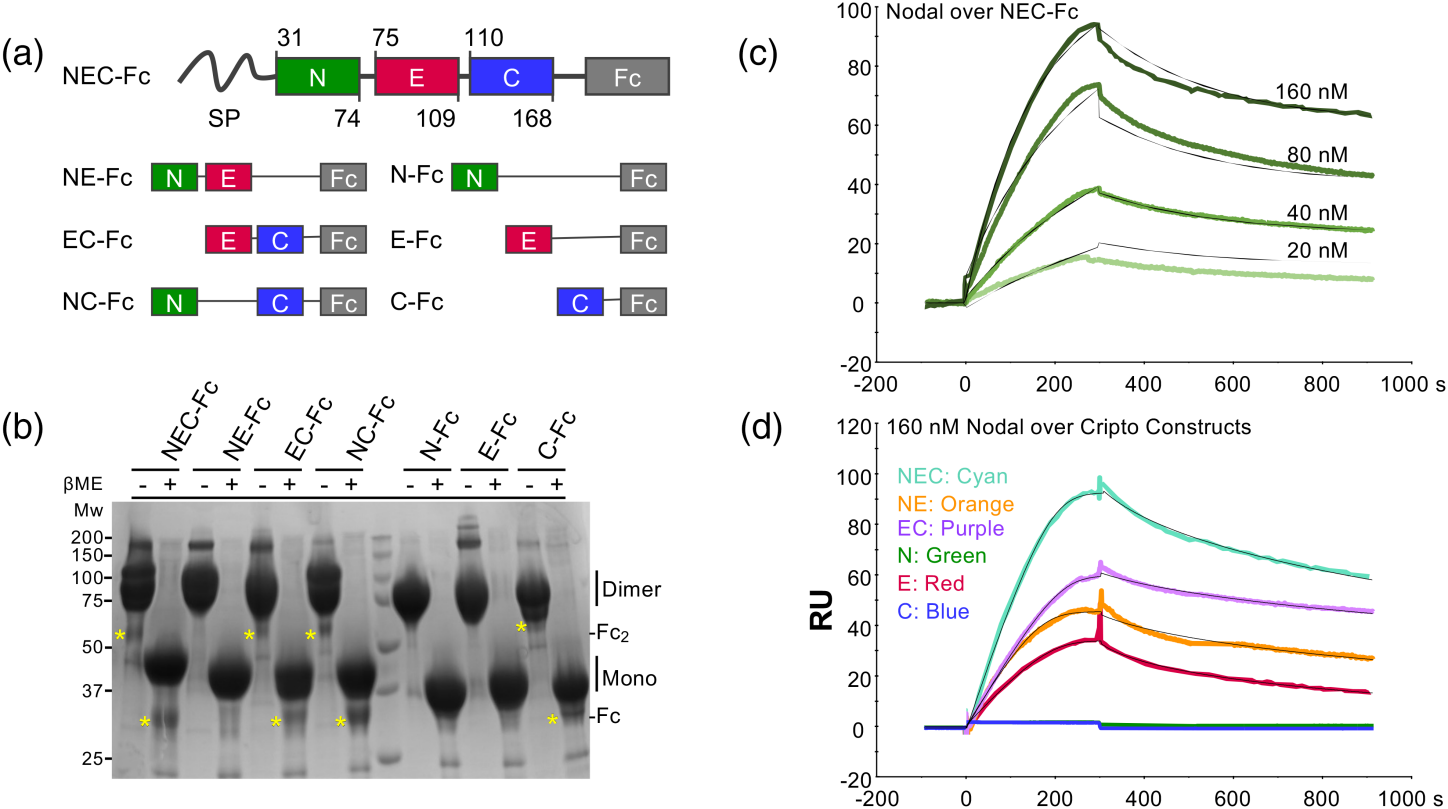
Design and validation of Cripto‐1 domain constructs for Nodal binding analysis. (a) Schematic representation of Cripto‐1 domain constructs fused to human IgG1 Fc. The N‐terminal (N) domain is shown in green, the EGF‐like (E) domain in red, and the CFC (C) domain in blue. Construct names, reflecting their composition, are shown next to the corresponding schematic. (B) SDS‐PAGE analysis of purified Cripto‐1‐Fc constructs under non‐reducing (left) and reducing (right) conditions. The yellow star indicates Fc fragments resulting from proteolytic cleavage. (C) SPR sensorgram depicting the binding of increasing concentrations of Nodal to immobilized full‐length Cripto‐1 (NEC‐Fc). Curves are color‐coded by Nodal concentration (nM), and modeled curves are shown as black lines. (D) Single‐concentration (160 nM) SPR analysis of Nodal binding to different Cripto‐1 domain constructs. Colors represent to distinct domain architectures shown in (a), that is, NEC‐Fc (cyan), NE‐Fc (orange), EC‐Fc (purple), N‐Fc (green), E‐Fc (red), and C‐Fc (blue).

To validate the AlphaFold3 model, we turned to SPR, a method that has become a cornerstone in the study of biomolecular interactions. We engineered various Cripto‐1 constructs consisting of domain combinations fused to human IgG1 Fc (Figure 3a). These included a soluble full‐length variant (NEC‐Fc) lacking the GPI anchor, constructs containing two domains (NE‐Fc, EC‐Fc, and NC‐Fc), and constructs containing single domains (N‐Fc, E‐Fc, and C‐Fc), where “N” denotes the N‐terminal domain, “E” the EGF‐like domain, and “C” the CFC domain. Each construct was expressed in Chinese hamster ovary (CHO) cells and purified to homogeneity using protein A affinity chromatography. Size exclusion chromatography (SEC) analysis of the recombinant Cripto‐1 Fc fusion proteins revealed predominantly monodisperse elution profiles. This observation is consistent with the non‐reducing SDS‐PAGE analysis, which showed that most of the purified protein migrated as the expected dimer, with a residual fraction present as higher‐order oligomers (Figure 3b). Although these findings suggest that the Cripto‐1 Fc fusion proteins are predominantly dimeric, we acknowledge that SEC and SDS‐PAGE may not fully detect heterogeneities in the folding of the small, disulfide‐rich Cripto‐1 domains within the larger Fc fusion context.

Using SPR, we confirmed the predicted interaction between Nodal and Cripto‐1, observing an apparent equilibrium dissociation constant (*K*_D_) of 1.01 × 10^−8^ M (Figure 3c). To further investigate the binding interaction, we conducted SPR studies with Nodal and the individual NE‐Fc, EC‐Fc, and E‐Fc domain constructs. These studies demonstrated that Nodal binds to all three constructs with comparable low nanomolar *K*_D_ values (Figures 3d, S3, and Table 1). The direct binding of Nodal to the isolated EGF‐like domain validates the AlphaFold3‐predicted interaction (Figure 2a) and highlights the central role of this domain in ligand recognition. It is important to note that both Nodal and the Fc‐fusion proteins used in these studies are dimeric, which could lead to an overestimation of the binding affinity due to avidity effects.

**TABLE 1:** Equilibrium dissociation and rate constants.

Capture	Analyte	*k*_a_ (M^−1^ s^−1^)	*k*_d_ (s^−1^)	*K*_D_ (M)	Chi^2^ (RU^2^)
Nodal binding to Cripto‐1					
NEC‐Fc	Nodal	1.14e6 ± 5.84e4	1.16e‐2 ± 4.03e‐4	1.01e‐8 ± 1.43e‐9	1.25
NE‐Fc		3.41e4 ± 202	1.04e‐3 ± 6.35e‐6	1.04e‐8 ± 8.85e‐11	1.25
EC‐Fc		2.82e4 ± 4.88e4	4.04e‐4 ± 2.51e‐4	1.43e‐8 ± 2.63e‐8	1.72
E‐Fc		1.95e5 ± 2.31e4	5.41e‐3 ± 3.43e‐4	2.78e‐8 ± 3.74e‐9	0.97
mAbs binding to Cripto‐1					
NEC‐Fc	N‐mAb	3.97e5 ± 9.69e4	6.93e‐6 ± 1.39e‐5	1.75e‐11 ± 4.10e‐11	49.6
Ne‐Fc		4.35e4 ± 1.29e3	1.04e‐7 ± 2.54e‐6	2.39e‐12 ± 6.14e‐11	3.20
N‐Fc		1.06e4 ± 6.58e2	6.42e‐4 ± 1.55e‐5	6.06e‐8 ± 9.18e‐10	7.58
NEC‐Fc	E‐mAb	2.77e5 ± 5.03e2	5.45e‐6 ± 2.87e‐7	1.97e‐11 ± 2.73e‐12	26.40
NE‐Fc		2.75e5 ± 1.26e3	1.51e‐5 ± 2.85e‐6	5.49e‐11 ± 1.45e‐11	22.70
EC‐Fc		2.68e5 ± 5.32e2	5.50e‐5 ± 2.17e‐7	2.05e‐10 ± 9.95e‐13	11.30
E‐Fc		5.49e5 ± 2.11e3	1.19e‐5 ± 7.31e‐7	2.16e‐11 ± 1.26e‐10	2.38
NeC‐Fc	C‐mAb	1.22e5 ± 6.00e2	1.62 e‐6 ± 1.07e‐7	1.33e‐11 ± 1.49e‐12	51.90
EC‐Fc		1.24e5 ± 1.12e3	6.51e‐5 ± 5.84e‐6	5.25e‐10 ± 5.12e‐11	4.51
C‐Fc		1.78e5 ± 7.29e2	3.00e‐5 ± 5.34e‐8	1.69e‐10 ± 7.55e‐13	32.00
ALK4‐Fc	NEC	2.24e4 ± 105	1.79e‐3 ± 1.15e‐5	7.99e‐8 ± 6.36e‐10	1.16

### 2.3 Domain‐specific antibodies elucidate individual Cripto‐1 functions

To complement the domain deletion strategy in elucidating the roles of Cripto‐1 domains, we developed domain‐specific monoclonal anti‐Cripto‐1 antibodies (mAbs). We immunized mice with the soluble Cripto‐1 NEC fragment. To assess the resulting hybridoma clones, we conducted a high‐throughput SPR screen. This involved capturing antibodies from the hybridoma supernatants on an SPR sensor chip, followed by the injection of Cripto‐1‐Fc (NEC‐Fc, Figure S4A). Through this initial screen, we identified anti‐Cripto‐1 mAbs with distinct kinetic profiles and assessed the productivity of the corresponding hybridoma clones. To further refine our selection and determine the domain binding specificities of the mAbs, we captured antibodies from the top‐performing hybridoma clones on the SPR sensor chip. We then flowed a panel of Cripto‐1‐Fc fusion proteins, each containing different domains of Cripto‐1, over the captured antibodies (Figure S4B–D). This approach allowed us to identify mAbs with distinct domain binding preferences. Based on this comprehensive analysis, we chose three mAbs (H3A6, H11N4, and G1H2) for further characterization, each exhibiting a strong binding affinity for Cripto‐1, as evidenced by their kinetic profiles, and each likely targeting a different Cripto‐1 domain.

**FIGURE 4:**
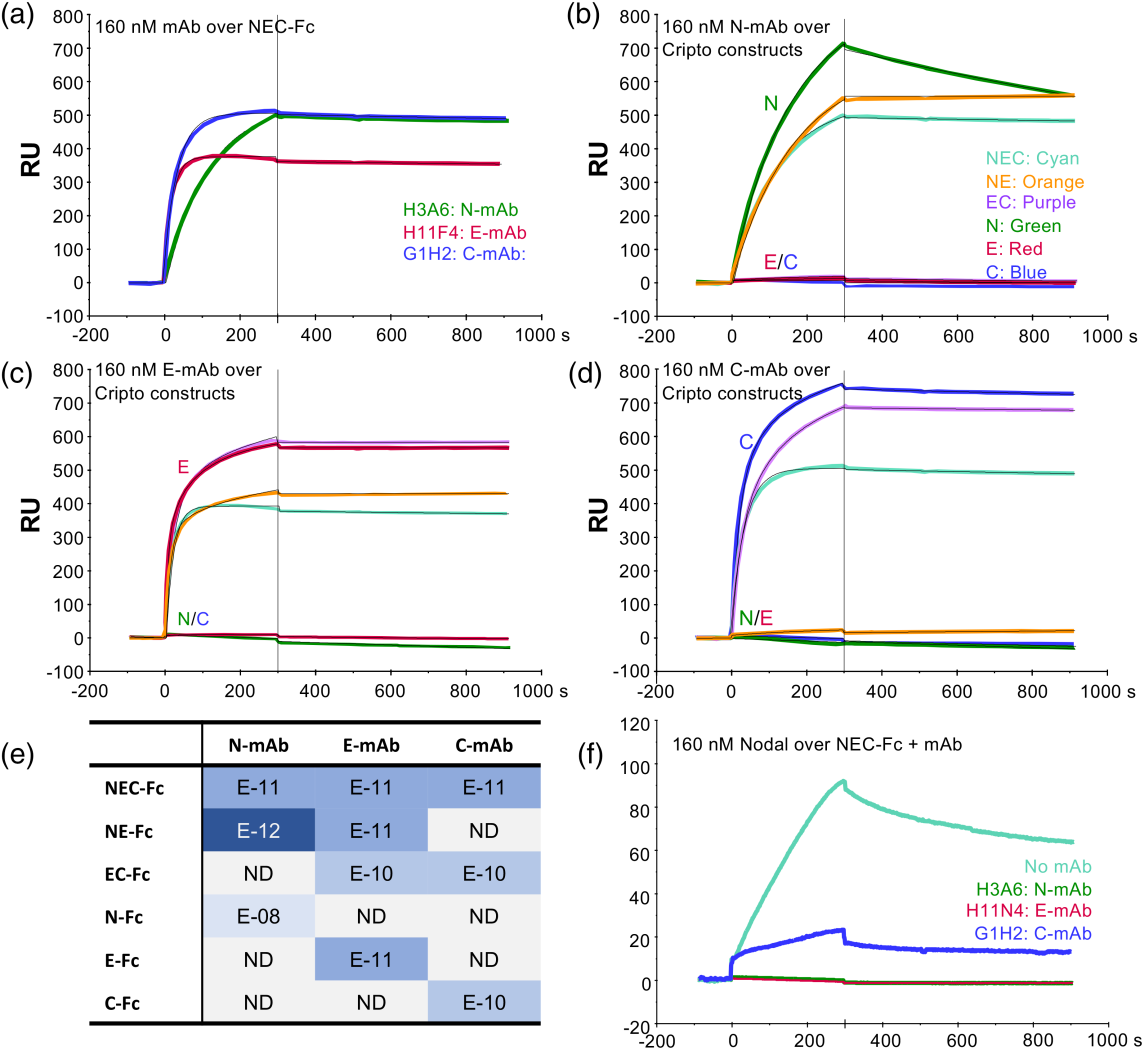
Characterization of domain‐specific anti‐Cripto‐1 monoclonal antibodies and their impact on Nodal binding. (a) SPR sensorgrams of single‐concentration (160 nM) injections of anti‐Cripto‐1 monoclonal antibodies (mAbs) over immobilized full‐length Cripto‐1‐Fc. Curves are color‐coded according to each mAb's binding epitope, as determined in panels B‐D: Green (H3A6, N‐domain), red (H11F4, EGF‐like domain), and blue (G1H2, CFC domain). (B)–(D) SPR analysis to map the binding epitope for each mAb. Different Cripto‐1 domain constructs (NEC‐Fc, NE‐Fc, EC‐Fc, N‐Fc, E‐Fc, and C‐Fc) were immobilized, and individual mAbs (160 nM) were injected over each surface. Sensorgrams are color‐coded as in panel A. (b) H3A6 binds the single domain construct N‐Fc (green curve) and is therefore designated as the N‐mAb. (c) H11F4 binds the single domain construct E‐Fc (red curve) and is therefore designated as the E‐mAb. (d) G1H2 binds the single domain construct C‐Fc (blue curve) and is, therefore, designated as the C‐mAb. (e) Heatmap depicting the equilibrium dissociation constants (*K*_D_ values) for each mAb binding to the different Cripto‐1 domain constructs. *K*_D_ values were determined from multi‐cycle kinetic titrations (sensorgrams not shown). Darker shades of blue indicate higher affinity interactions. NB = no binding detected. (f) SPR analysis of the effect of mAbs on Nodal binding to Cripto‐1. Full‐length Cripto‐1‐Fc was captured on an anti‐human Fc surface. Individual mAbs (1 μM) were captured first, followed by Nodal (160 nM). Nodal binds Cripto‐1‐Fc in the absence of mAb (cyan curve). The C‐mAb partially blocks Nodal binding (blue curve), while the N‐mAb (green curve) and E‐mAb (red curve) completely inhibit the Nodal‐Cripto‐1 interaction.

To confirm the specificity of the selected mAbs, we immobilized various Cripto‐1 constructs, including single‐domain Fc‐fusion proteins (N‐Fc, E‐Fc, and C‐Fc), on an SPR sensor chip and flowed the purified antibodies over the surface (Figure 4a–e). Our results demonstrate that each mAb binds exclusively to a distinct domain: H3A6 (N‐mAb) to the N‐terminal domain, H11N4 (E‐mAb) to the EGF‐like domain, and G1H2 (C‐mAb) to the CFC domain. This domain specificity was confirmed by the selective binding of each mAb to the corresponding single‐domain Cripto‐1 construct (Figure 4a–e, S5–S7). Notably, the E‐mAb and C‐mAb retained binding affinities to their isolated domains comparable to full‐length Cripto‐1 (Table 1), indicating that these domains maintain their structural integrity when expressed individually. In contrast, the N‐mAb exhibited a faster dissociation rate from the isolated N‐domain construct, suggesting that the context of the full‐length Cripto‐1 protein contributes to the N‐mAb binding. This contribution could involve interactions with other domains or be due to residues within the N‐domain that are immediately adjacent to the EGF domain and may be affected by the domain boundary in the isolated construct. The unique specificity of these three mAbs offers a powerful toolkit to dissect the enigmatic function of Cripto‐1 and explore its complex interactions in development and disease.

To investigate how these domain‐specific mAbs might affect the interaction between Cripto‐1 and ligands, we used SPR to assess their effects on Nodal binding. We immobilized Cripto‐1–mAb complexes on an SPR sensor chip, then flowed Nodal at 160 nM over the surface (Figure 4f). AlphaFold3 predicted a role for the EGF‐like domain in Nodal binding. Consistent with this prediction, the E‐mAb completely abrogated Nodal binding. This was evidenced by the absence of a detectable Nodal SPR signal after we preincubated Cripto‐1 with the E‐mAb. The N‐domain mAb also inhibited the interaction between Cripto‐1 and Nodal, supporting the AlphaFold3 prediction that the C‐terminal residues of the N‐domain contribute to Nodal binding (Figure 2b). In contrast, AlphaFold3 did not predict an interaction between the C‐domain and Nodal. Nevertheless, we observed partial inhibition with the C‐mAb. We speculate that this likely results from sterically blocking Nodal access to its binding site on the EGF‐like domain. Alternatively, the C‐mAb might induce conformational changes in Cripto‐1 through an allosteric mechanism that disrupts or masks the Nodal binding interface.

### 2.4 Interaction analysis validates Cripto‐1 association with ALK4

**FIGURE 5:**
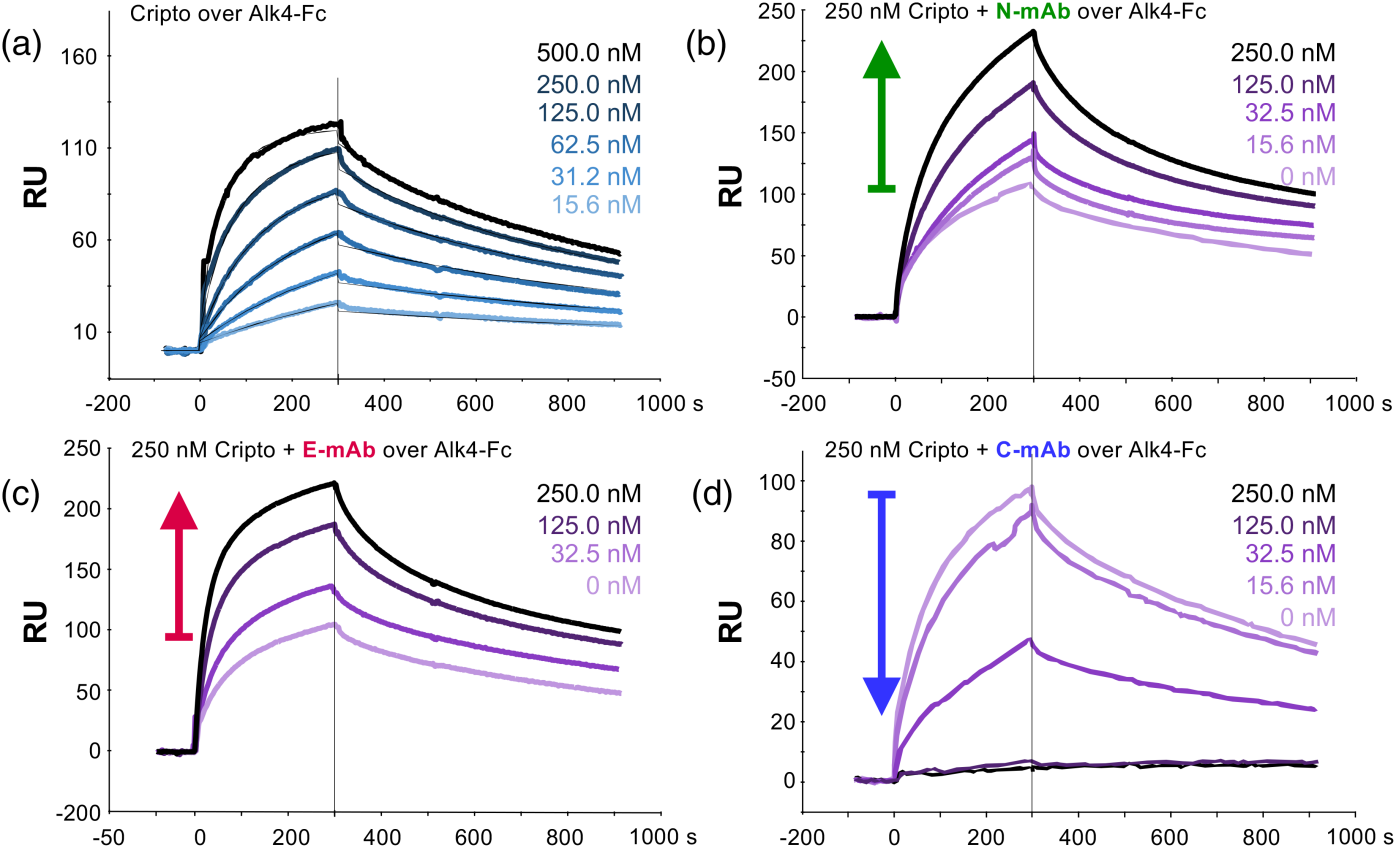
Analysis of Cripto‐1 binding to the ALK4 ligand‐binding domain and the effect of domain‐specific antibodies. (a) SPR sensorgram depicting the binding of increasing concentrations of Fc‐free Cripto‐1 to immobilized ALK4 ligand‐binding domain (ALK4‐Fc). Each curve represents a different concentration of Cripto‐1, as indicated in the legend. Modeled curves are shown as black lines. (b)‐(d) SPR analysis to determine the effect of domain‐specific anti‐Cripto‐1 mAbs on the interaction between Cripto‐1 and ALK4. ALK4‐Fc was captured on the sensor chip, and a constant concentration (250 nM) of Fc‐free Cripto‐1 was pre‐incubated with increasing concentrations of each mAb. The resulting sensorgrams show the change in SPR response upon injection of the Cripto‐1/mAb mixture compared to the mAb alone, reflecting the impact of the mAb on Cripto‐1 binding to ALK4. (b) Titration of the N‐mAb (green sensorgrams, arrow indicates increasing SPR response with increasing N‐mAb concentration from 0 to 250 nM), suggesting the formation of a higher molecular weight Cripto‐1–N‐mAb complex that retains the ability to bind ALK4. (c) Titration of the E‐mAb (red sensorgrams, arrow indicates increasing SPR response with increasing E‐mAb concentration from 0 to 250 nM), suggesting the formation of a higher molecular weight Cripto–E‐mAb complex that retains the ability to bind ALK4. (d) Titration of the C‐mAb (blue sensorgrams, arrow indicates decreasing SPR response with increasing C‐mAb concentration from 0 to 250 nM), indicating that the C‐mAb inhibits the Cripto‐1/ALK4 interaction in a dose‐dependent manner.

Previous co‐IP studies (Gray et al., 2003; Yeo & Whitman, 2001) suggest an interaction between Cripto‐1 and the type I receptor ALK4, and our AlphaFold3 modeling supports this hypothesis (Figure 1e, f). However, this interaction has not yet been demonstrated using purified Cripto‐1 and ALK4. To directly test the interaction between these two proteins, we investigated their binding using SPR. We captured the Fc‐fused ALK4 ligand‐binding domain on an SPR sensor chip and injected varying concentrations of Cripto‐1, from which the Fc fragment had been proteolytically removed, over the surface (Figure 5a). Analysis of the resulting SPR sensorgram demonstrates direct binding of Cripto‐1 to ALK4, with an equilibrium binding constant (*K*_D_) of 7.99 × 10^−8^ M. Notably, our previous reverse approach, capturing Cripto‐1‐Fc on a sensor chip and flowing Fc‐free ALK4 over the chip, did not give clear results (Aykul et al., 2017). This likely resulted from partial loss of ALK4 activity due to destabilization or misfolding after proteolytic removal of the Fc domain, affecting its binding capability. However, the robust binding observed in our current experiments overcomes the limitations of our previous approach and strongly supports a direct, high‐affinity interaction between Cripto‐1 and the ALK4 ligand‐binding domain.

To identify the Cripto‐1 domain responsible for ALK4 binding, we utilized our domain‐specific mAbs in a series of SPR experiments. We preincubated 160 nM Fc‐free Cripto‐1 with increasing concentrations of each mAb, then flowed these complexes over captured ALK4‐Fc. Both the N‐ and E‐mAbs elicited a dose‐dependent increase in the SPR response, suggesting that these Cripto‐1–mAb complexes bind to ALK4 (Figure 5b, c). This dose‐dependent increase in SPR response likely reflects the binding of larger Cripto‐1–mAb complexes. These findings indicate that the N‐ and E‐domains do not directly mediate the interaction with ALK4, as mAb binding to these domains did not disrupt Cripto‐1–ALK4 complex formation. In contrast, the anti‐CFC mAb led to a dose‐dependent decrease in the SPR signal (Figure 5d). This inhibition demonstrates that the CFC domain is responsible for mediating Cripto‐1/ALK4 binding.

**FIGURE 6:**
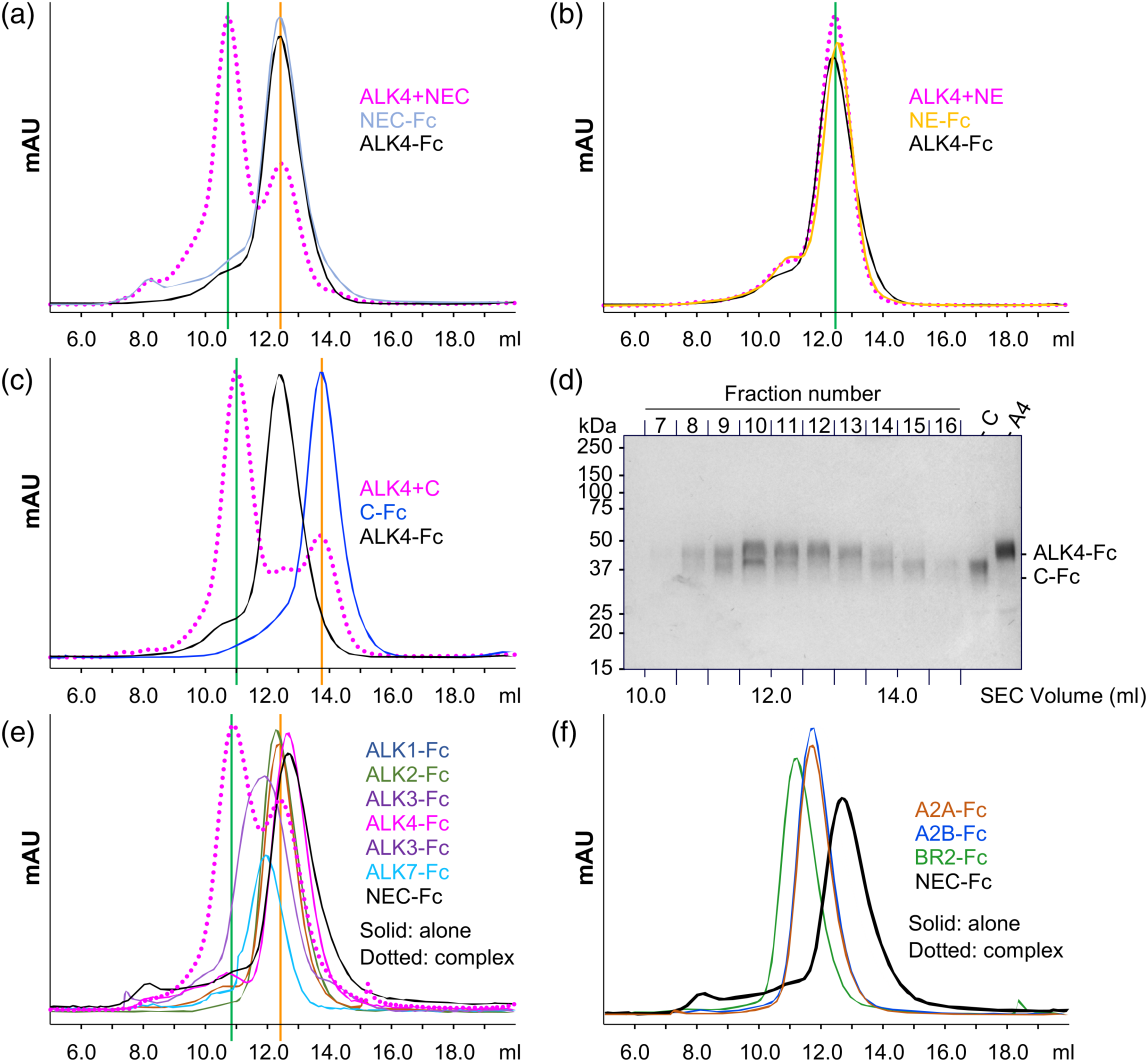
Analysis of the Cripto‐1 Interaction with ALK4 by size exclusion chromatography (SEC). (a) SEC chromatograms of ALK4‐Fc alone (black), full‐length Cripto‐1‐Fc (NEC‐Fc, light blue), and the ALK4‐Fc–Cripto‐1‐Fc complex (magenta). A clear shift in elution volume is observed for the complex, which elutes at approximately 10.75 mL, compared to the individual proteins, indicating the formation of a higher molecular weight complex. (b) SEC chromatograms of ALK4‐Fc alone (black), the EGF‐CFC domains of Cripto‐1 fused to Fc (NE‐Fc, orange), and a mixture of ALK4‐Fc and NE‐Fc (magenta). No shift in elution volume is observed for ALK4‐Fc plus NE‐Fc, suggesting that the NE‐Fc construct does not form a complex with ALK4. (c) SEC chromatograms of ALK4‐Fc alone (black), the CFC domain of Cripto‐1 fused to Fc (C‐Fc, blue), and the pre‐formed ALK4‐Fc–C‐Fc complex (magenta). A clear shift in elution volume is observed for the complex compared to the individual proteins, suggesting that the CFC domain of Cripto‐1 is sufficient for interaction with ALK4. (d) SDS‐PAGE analysis under reducing conditions of the main peak fractions (10–15 mL) from SEC of the C‐Fc–ALK4‐Fc complex shows co‐elution of both proteins. C‐Fc (C) and ALK4‐Fc (A4) alone were run as controls to identify the respective bands. (e) SEC chromatograms of type I receptors alone (solid lines) and in complex with full‐length Cripto‐1‐Fc (NEC‐Fc, dotted lines). The chromatograms correspond to ALK1‐Fc (dark blue), ALK2‐Fc (green), ALK3‐Fc (purple), ALK4‐Fc (magenta), and ALK7‐Fc (cyan). Cripto‐1‐Fc alone is shown in black. Only ALK4‐Fc shows a shift in elution volume when combined with Cripto‐1‐Fc, indicating specific binding. (f) SEC chromatograms of type II receptors alone (solid lines) and in complex with full‐length Cripto‐1‐Fc (NEC‐Fc, dotted lines). The chromatograms correspond to ACVR2A‐Fc (A2A, orange), ACVR2B‐Fc (A2B, blue), and BMPR2‐Fc (BR2, green). None of the tested type II receptors exhibit a shift in elution volume when combined with Cripto‐1‐Fc, suggesting that Cripto‐1 does not interact with these receptors.

Having established a direct interaction between Cripto‐1 and ALK4 by SPR, we next sought to map the ALK4 binding site on Cripto‐1 using SEC and our Cripto‐1 domain deletion constructs. Combining full‐length Cripto‐1‐Fc with ALK4‐Fc resulted in a complex that eluted with a significantly earlier volume (10.5 mL) compared to that of either protein alone (approximately 13 mL) (Figure 6a). Notably, the NE‐Fc fragment (lacking the CFC domain) showed no shift in elution volume when combined with ALK4‐Fc, indicating that the N and E domains do not bind ALK4 (Figure 6b). Conversely, the C‐Fc construct (containing only the CFC domain) showed a marked shift to an earlier elution volume (11 mL) when combined with ALK4‐Fc, compared to 13.75 mL for C‐Fc alone, confirming that this domain is sufficient for complex formation (Figure 6c). We further confirmed this interaction by SDS‐PAGE analysis, which showed co‐elution of ALK4‐Fc and the C‐Fc construct in higher molecular weight fractions, supporting the specific binding of ALK4 to the CFC domain (Figure 6d).

To explore potential TGF‐*β* family receptors that interact with Cripto‐1, we tested the binding of various purified receptor–Fc fusion proteins to full‐length Cripto‐1–Fc (NEC‐Fc). Among the diverse type I and type II receptors tested, only ALK4 induced a shift in the elution volume of NEC‐Fc (Figure 6e, f), highlighting the specificity of this interaction. Further supporting this specificity, our sequence alignment and structural analysis of the ALK4‐Cripto‐1 interface (Figure S8) shows that the Cripto‐1‐contacting residues in ALK4 are not conserved in other type I receptors, suggesting a unique binding mode. Taken together, our SPR and SEC data provide strong evidence that the CFC domain is the primary mediator of the specific interaction between Cripto‐1 and ALK4. This specific interaction may have important implications for the distinct signaling outcomes mediated by Cripto‐1.

### 2.5 Signaling modulation by Cripto‐1 in NTERA‐2 cells

**FIGURE 7:**
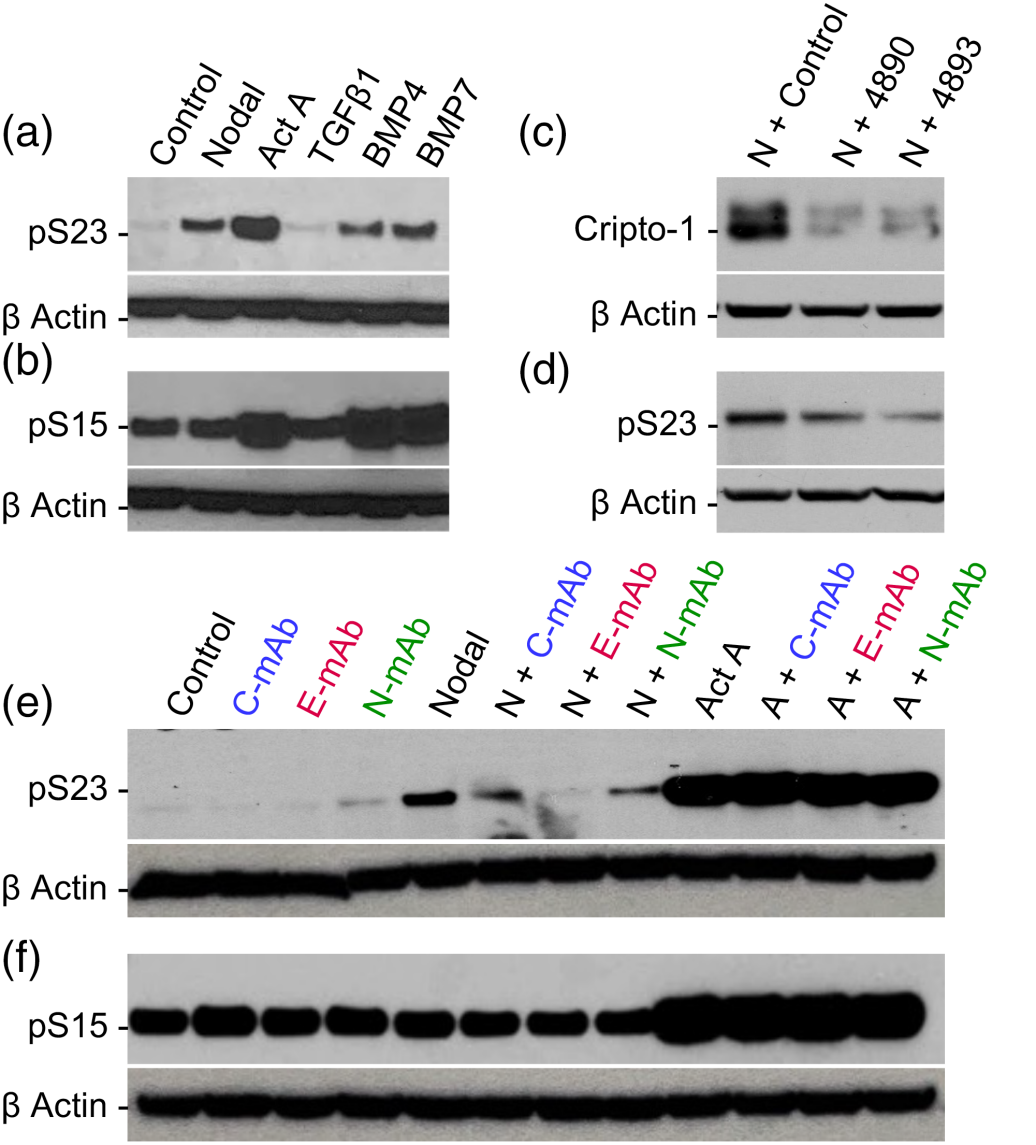
Cripto‐1 regulates Nodal‐induced SMAD2/3 signaling in NTERA‐2 cells. (a) Western blot analysis of phosphorylated SMAD2/3 (pS23) levels in NTERA‐2 cells treated with various TGF‐*β* family ligands (4 nM Nodal, 0.2 nM Activin A, 0.2 nM TGF‐*β*1, 0.2 nM BMP‐4, or 0.2 nM BMP‐7) for 1 h. *β*‐actin serves as a loading control. (b) Western blot analysis of phosphorylated SMAD1/5/8 (pS15) levels in the same samples as in (a). *β*‐actin serves as a loading control. (c) Western blot analysis confirming shRNA‐mediated knockdown of Cripto‐1 expression in NTERA‐2 cells. Scrambled shRNA was used as a control. Individual clone IDs are indicated, and Nodal (4 nM, 1 h) treatment is indicated by “N.” *β*‐actin serves as a loading control. (d) Western blot analysis of pSMAD2/3 levels induced by Nodal (4 nM) treatment for 1 h in control (scrambled shRNA) and Cripto‐1 knockdown NTERA‐2 cells (samples are the same as in c). *β*‐actin serves as a loading control. (e) Western blot analysis of pSMAD2/3 (pS23) levels in NTERA‐2 cells treated with Nodal (4 nM) or Activin A (0.2 nM) for 1 h, with or without the addition of 300 ng/mL of each anti‐Cripto‐1 antibody (N‐mAb, E‐mAb, or C‐mAb). Nodal treatment is indicated by “N” and Activin A treatment is indicated by “A.”. *β*‐actin serves as a loading control. (f) Western blot analysis of pSMAD1/5/8 (pS15) levels in NTERA‐2 cells treated as described in (e). *β*‐actin serves as a loading control.

To translate our biochemical findings into a cellular context, we employed the Cripto‐1‐expressing NTERA‐2 cell line, a pluripotent human testicular embryonic carcinoma cell line that exhibits similar properties to embryonic stem cells (Dono et al., 1991; Watanabe et al., 2010). While the response to TGF‐*β* family ligands can vary significantly depending on the cell type, our initial characterization of NTERA‐2 cells revealed a unique pattern of SMAD activation. Specifically, Nodal and Activin A‐induced robust SMAD2/3 phosphorylation, while TGF‐*β*1 did not (Figures 7a, S9). Interestingly, BMP‐4 and BMP‐7 also induced SMAD2/3 signaling in these cells. In contrast, we observed elevated basal SMAD1/5/8 phosphorylation, which was further enhanced by BMP‐4 and BMP‐7, as expected. Notably, Activin A also induced SMAD1/5/8 signaling in NTERA‐2 cells, but not Nodal, potentially highlighting a critical distinction between these two ligands in this cellular context (Figure 7b).

To directly assess the role of Cripto‐1 in Nodal signaling, we performed shRNA‐mediated Cripto‐1 knockdown in NTERA‐2 cells (Figure 7c). As expected, given its established role as an obligate Nodal co‐receptor, Cripto‐1 knockdown led to a reduction in Nodal‐induced SMAD2/3 phosphorylation, as indicated by the decreased p‐SMAD2/3 signal in Western blots (Figure 7d). This aligns with previous studies that indicated the requirement of Cripto‐1 for Nodal signaling in various cellular contexts (Schiffer et al., 2001; Yeo & Whitman, 2001). To analyze the function of Cripto‐1 domains in Nodal signaling, we tested our domain‐specific mAbs on NTERA‐2 cells. As anticipated, all three mAbs inhibited Nodal signaling, as evidenced by reduced p‐SMAD2/3 levels in Western blots (Figure 7e). While the E‐mAb and N‐mAb bind distinct Nodal interacting sites, inhibition by the C‐mAb likely results from its ability to disrupt Cripto‐1–ALK4 binding. Notably, these mAbs did not affect p‐SMAD2/3 levels in response to Activin A, indicating that Cripto‐1 does not directly participate in Activin A signaling in this context. Anti‐Cripto‐1 mAbs also did not inhibit SMAD1/5/8 phosphorylation, as evidenced by unchanged p‐SMAD1/5/8 levels in response to various ligands, including Nodal and Activin A (Figure 7f). This is likely because Cripto‐1 does not interact with receptors that activate the SMAD1/5/8 pathway. These findings highlight the specific role of Cripto‐1 as an essential co‐receptor for Nodal‐mediated SMAD2/3 activation while confirming its lack of involvement in Activin A‐ and BMP‐mediated signaling.

## 3 DISCUSSION

In this study, we elucidated the molecular mechanism of Cripto‐1‐mediated Nodal signaling using an integrative approach that combined AlphaFold3 modeling, protein engineering, antibody development, biochemical analysis, and cellular assays. Our findings indicate that Cripto‐1 functions as a molecular bridge between Nodal and its receptors, revealing a key distinction between Nodal signaling and other TGF‐*β* pathways.

### 3.1 Cripto‐1: A molecular bridge linking ligands and receptors

**FIGURE 8:**
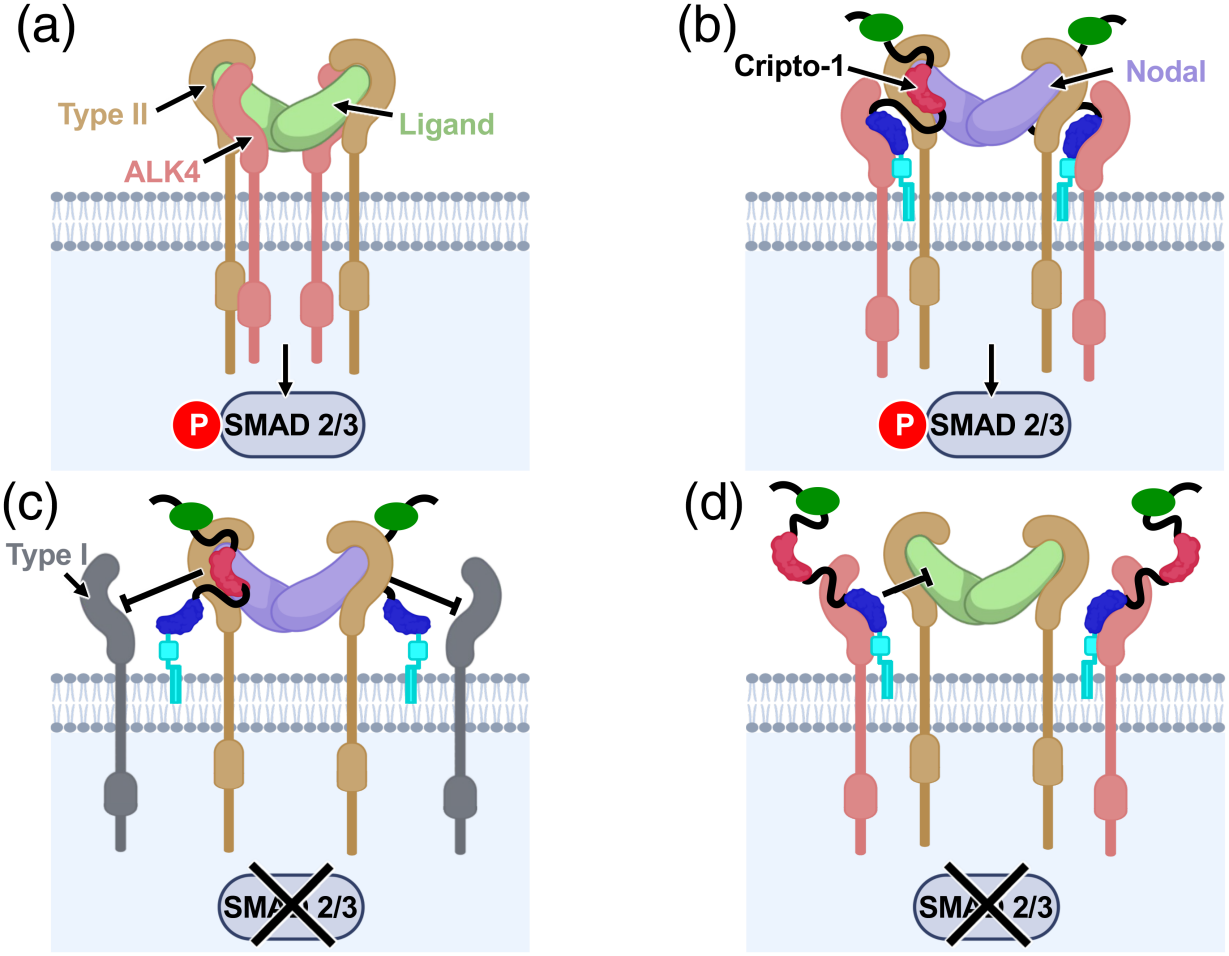
Schematic representation of Cripto‐1's role in modulating TGF‐*β* signaling. (a) Canonical TGF‐*β* signaling complex. A typical TGF‐*β* ligand (light green) binds directly to a type II receptor (gold), which then recruits and activates a type I receptor (salmon). This complex initiates downstream signaling by phosphorylating SMAD2/3 or SMAD1/5/8 transcription factors. (b) Cripto‐1‐dependent TGF‐*β* signaling complex. The ligand Nodal (light blue) likely does not directly bind the type I receptor ALK4 (salmon). Instead, the co‐receptor Cripto‐1 acts as a bridge, with its EGF‐like domain (red) binding Nodal and its CFC domain (blue) binding ALK4, enabling the formation of a ligand‐receptor complex that activates SMAD2/3 signaling. The N‐terminal domain of Cripto‐1, for which structural information is unavailable, is represented as a green circle. The Cripto‐1 GPI membrane anchor is schematically illustrated as the cyan colored shape. (c) Cripto‐1 antagonism via ligand sequestration. In this scenario, Cripto‐1 (with the EGF‐like domain in red) binds and sequesters Nodal (light blue) or other Cripto‐1‐binding ligands, preventing their interaction with type I receptors (gray) that do not bind Cripto‐1. This inhibits downstream signaling. (d) Cripto‐1 antagonism via receptor Blockade. In this scenario, Cripto‐1 (with the CFC domain in blue) binds to the type I receptor ALK4 (salmon) but is unable to recruit a ligand‐type II receptor complex if the ligand doesn't bind Cripto‐1. This blocks the ligand‐binding site on ALK4, preventing the formation of the signaling complex and inhibiting downstream signaling.

TGF‐*β* family signaling typically follows a canonical mechanism in which dimeric ligands bind directly to both type I and type II receptors, forming a complex with a 2:2:2 stoichiometry to activate downstream signaling. However, the TGF‐*β* family ligand Nodal deviates from this paradigm by requiring Cripto‐1 for signaling. How Cripto‐1 enables Nodal signaling is not known. To address this question, we used AlphaFold3 to predict the structure of a putative Cripto‐1–Nodal–ALK4–ACVR2B signaling complex. Our model provides the first structural insights into how Cripto‐1 acts as a molecular bridge that binds to Nodal via its EGF‐like domain and to the type I receptor ALK4 via its CFC domain (Figure 8). This brings ALK4 into proximity with the Nodal‐bound type II receptor, ACVR2B, facilitating the formation of a signaling complex that mirrors the canonical 2:2:2 stoichiometry typical of TGF‐*β* family complexes.

The Cripto‐1 bridging function ensures Nodal signaling specificity. Unlike other TGF‐*β* family ligands, such as Activin A and B, which can bind directly to both type I and type II TGF‐*β* family receptors, Nodal requires Cripto‐1 to effectively engage with both receptor types. This fundamental difference in receptor engagement restricts Nodal signaling to specific contexts where Cripto‐1 is expressed, allowing for precise spatiotemporal control of Nodal activity, such as defining the site of Nodal signaling in the early embryo.

While other TGF‐*β* co‐receptors participate in signaling modulation, they employ distinct mechanisms. For example, the repulsive guidance molecules (RGMs) are GPI‐anchored co‐receptors that enhance BMP signaling. While RGMb directly binds to BMP ligands like BMP‐2 and BMP‐4, it does not bridge the ligand and type I receptor as Cripto‐1 does. Instead, RGMs promote receptor assembly and complex stability in lipid rafts, influencing receptor localization (Bell et al., 2013; Healey et al., 2015). Similarly, co‐receptors like betaglycan and endoglin modulate TGF‐*β* signaling by altering ligand binding or facilitating receptor complex assembly, but they lack the direct bridging function of Cripto‐1. Betaglycan, for instance, binds TGF‐*β* homodimers asymmetrically, promoting TβRII binding but not engaging in the bridging seen with Cripto‐1. (Kim et al., 2019). Endoglin, on the other hand, selectively binds BMP‐9 and BMP‐10, enhancing their signaling through a selective ligand recognition mechanism (Castonguay et al., 2011; Saito et al., 2017). Therefore, the dual binding of Cripto‐1 to both Nodal and ALK4 represents a unique mechanism within the TGF‐*β* family, highlighting its specialized role in Nodal signaling.

### 3.2 Validating the AlphaFold3 model

We employed SPR to validate the interactions predicted by AlphaFold3. Our results confirmed high‐affinity binding between Nodal and all Cripto‐1 constructs containing the EGF‐like domain, establishing this domain as the core Nodal binding site. Our AlphaFold3 model further identified the fucosylated threonine 88 (T88) within this domain as a key contact residue. This prediction is consistent with previous studies demonstrating that mutation of T88 disrupts Nodal signaling (Schiffer et al., 2001). The conservation of residues contacting the fucosylated T88 in BMPs, but not Activins, further supports the accuracy of the AlphaFold3 model and provides a rationale for the observed ligand binding specificity of Cripto‐1. Notably, the AlphaFold3 model also predicted that Cripto‐1 binds Nodal at an epitope typically occupied by type I receptors in other TGF*‐β* ligand‐receptor complexes. This prediction is consistent with our earlier SPR data, which demonstrated that Cripto‐1 inhibits the interaction between the type I receptor ALK3 and its ligand BMP‐4 (Aykul et al., 2017). Notably, the PNPVG sequence in Nodal contacted by the fucosylated T88 is not conserved in Activin A (Figure S10), which may explain why Cripto‐1 does not bind Activin A. However, this sequence has some conservation in BMP‐4, consistent with the observed binding of this ligand to Cripto‐1. This conservation in BMP‐4, but not Activins, provides a rationale for the observed ligand binding specificity of Cripto‐1 (Aykul et al., 2017).

SPR analysis also confirmed the predicted interaction between Cripto‐1 and ALK4 via the CFC domain. This finding was further supported by SEC experiments, which demonstrated that the formation of a higher molecular weight complex between Cripto‐1 and ALK4 requires the CFC domain. Importantly, the AlphaFold3 model predicted the positioning of the conserved tryptophan at position 123 (W123) in the CFC domain at the ALK4 interface. This prediction is consistent with previous studies showing that mutation of W123 disrupts ALK4 binding (Yeo & Whitman, 2001). In contrast to the formation of a complex between Cripto‐1 and the type I receptor ALK4, we did not observe binding between Cripto‐1 and other type I receptors, including ALK7, or with any type II receptors, highlighting the specificity of the Cripto‐1–ALK4 complex. The observed receptor‐binding specificity, coupled with the conservation of key interacting residues within the Cripto‐1–ALK4 complex, strongly suggests that Cripto‐1 uniquely facilitates Nodal signaling through ALK4.

To further investigate the interaction between Nodal and Cripto‐1, we developed monoclonal antibodies (mAbs) targeting specific Cripto‐1 domains. The mAb targeting the EGF‐like domain completely blocked Nodal binding, confirming the essential role of this domain in the Nodal–Cripto‐1 complex. Interestingly, the antibody targeting the N‐terminal domain also inhibited Nodal–Cripto‐1 binding. This observation is consistent with our AlphaFold3 predictions that show residues in the C‐terminal region of the N‐domain, positioned near the EGF‐like domain, directly contact Nodal. Although the EGF‐like domain is established as the primary site for ligand recognition, our findings suggest that the N‐domain may play a supporting role in this process. Furthermore, we also confirmed interaction between the CFC domain and ALK4 using an antibody targeting the CFC domain, which specifically blocked binding of Cripto‐1 to ALK4. Taken together, these findings expand our understanding of how multiple Cripto‐1 domains cooperate to mediate its function as a Nodal co‐receptor.

### 3.3 Functional relevance in NTERA‐2 cells

To assess the functional consequences of these interactions, we utilized NTERA‐2 cells, a human embryonal carcinoma cell line that endogenously expresses Cripto‐1 (Dono et al., 1991; Watanabe et al., 2010). Nodal signaling has historically been challenging to study due to the limited number of cell lines that respond robustly to this ligand. However, we found that recombinant Nodal consistently induced SMAD2/3 phosphorylation, the canonical readout of Nodal pathway activation, in NTERA‐2 cells. This robust and specific response positions NTERA‐2 cells as a valuable model system for investigating Nodal signaling and the function of Cripto‐1.

shRNA‐mediated knockdown of Cripto‐1 in NTERA‐2 cells attenuated Nodal‐induced SMAD2/3 phosphorylation, confirming its essential role as an obligate Nodal co‐receptor (Bianco et al., 2002; Yeo & Whitman, 2001). Providing further experimental validation of the biochemical interactions we identified, our domain‐specific antibodies significantly suppressed Nodal‐induced SMAD2/3 phosphorylation. This inhibition was accomplished by either preventing Nodal from binding to Cripto‐1 using the N‐ and E‐mAbs or by disrupting the interaction between Cripto‐1 and ALK4 using the C‐mAb.

Interestingly, Activin A activated both the SMAD2/3 and SMAD1/5/8 pathways in NTERA‐2 cells and anti‐Cripto‐1 antibodies failed to inhibit Activin A signaling. This result highlights distinct signaling preferences for Activin A and Nodal, despite their frequent interchangeable use in stem cell studies. Thus, Nodal, through its interaction with Cripto‐1, appears to be a specific activator of SMAD2/3 signaling among TGF‐*β* ligands in NTERA‐2 cells. This specificity may be crucial for the distinct developmental roles of Nodal compared to other TGF‐*β* ligands in stem cell fate determination.

### 3.4 Potential Cripto‐1 dual function

Beyond its role as a Nodal co‐receptor, Cripto‐1 may also function as an inhibitor of certain TGF‐*β* family ligands (Gray et al., 2003; Yan et al., 2002). Consistent with the AlphaFold3 model, our previous work demonstrated that Cripto‐1 can bind ligands at their type I receptor interface, potentially inhibiting their signaling (Aykul et al., 2017). Furthermore, AlphaFold3 models predict that Cripto‐1 interacts with the ligand‐binding site on ALK4, potentially preventing other ligands from binding to this receptor. Through these competitive interactions, Cripto‐1 could fine‐tune signaling by certain TGF‐*β* family members, acting as a context‐dependent antagonist. Nevertheless, direct experimental evidence for these inhibitory effects is currently limited, and further studies are needed to confirm and elucidate these potential mechanisms.

Despite these insights, several questions regarding the Cripto‐1‐mediated signaling mechanism remain unresolved. While our study focused on the initial assembly of the Cripto‐1–Nodal–receptor complex, it is possible that subsequent endocytosis of this complex modulates the duration or intensity of Nodal signaling, a process that warrants further investigation (Blanchet et al., 2008). Additionally, Cripto‐1 has been implicated in GDF‐8 (myostatin) signaling, promoting satellite cell activation and muscle hypertrophy by antagonizing GDF‐8 (Guardiola et al., 2012; Guardiola et al., 2023; Prezioso et al., 2015). Understanding the involvement of Cripto‐1 in GDF‐8 signaling could reveal new mechanisms by which it influences tissue‐specific functions and developmental processes.

## 4 CONCLUSIONS

Our study provides new insights into the mechanism of Cripto‐1 function in Nodal signaling. By acting as a molecular bridge between Nodal and ALK4, Cripto‐1 facilitates the assembly of a unique signaling complex that departs from the canonical TGF‐*β* signaling paradigm. The AlphaFold3 structural model, validated by extensive biochemical and cell‐based assays, explains the obligate co‐receptor role of Cripto‐1 in Nodal signaling and suggests a potential mechanism for its context‐dependent antagonism of certain other TGF‐*β* family ligands. This work highlights the power of integrating computational modeling with experimental validation to elucidate complex molecular mechanisms.

## 5 EXPERIMENTAL PROCEDURES

### 5.1 Expression plasmids

A synthetic *Cripto‐1‐hIgG‐Fc* gene was obtained from GeneArt. The full‐length fusion construct included the human Cryptic signal peptide (1–25) and the extracellular domains of human Cripto‐1 (31–163) linked to human IgG1 Fc via a 22‐amino acid linker containing a tobacco etch virus (TEV) cleavage site, a glycine/serine‐rich region, and a FLAG tag. Domain deletion constructs were generated by PCR or purchased from GeneArt. Ligands were produced in‐house (Activin A, Activin B, TGF‐*β*1) or purchased from RnD Systems (BMP‐4, BMP‐7, Nodal). We cloned the *Cripto‐1‐hIgG‐Fc* gene and the domain deletion constructs into the BstZ17I/AvrII restriction sites of the *p*CHO 1.0 expression vector.

### 5.2 Cell lines

CHO‐S cells were obtained from Thermo Fisher Scientific and grown in suspension culture using Dynamis CHO Expression Medium supplemented with 8 mM L‐glutamine and 5 mL/L of Penicillin/Streptomycin (0.5X). Following transfection, stable cell pools were selected by culturing the cells in medium containing puromycin and methotrexate to ensure the maintenance of the expression plasmid. Cells were maintained in a humidified atmosphere of 8% CO_2_ in air on an orbital shaker platform rotating at 125 rpm. Cultures were passaged every 3–4 days and seeded at a density of 3 × 10^5^ viable cells/mL.

NTERA‐2 cl.D1 (NT2/D1) cells (CRL‐1973) were obtained from American Type Culture Collection and grown in DMEM modified to contain 2 mM L‐glutamine, 4500 mg/L glucose, and 10% fetal bovine serum. The medium was supplemented with 1% Penicillin/Streptomycin. Cells were maintained at 37°C in a humidified atmosphere of 5% CO_2_ in air. Cultures were prepared by scraping and seeding new flasks at a density of at least 5 × 10^6^ viable cells per 75 cm^2^ flask. Medium was renewed every 2–3 days. ± NT2/D1 cells were passaged at least two times before performing assays, and passage number did not exceed 10.

### 5.3 Protein purification

Proteins were expressed using stably transfected CHO‐S cell pools. Cells were cultured in suspension using Dynamis CHO Expression Medium supplemented with 8 mM L‐glutamine. Cells were maintained in a humidified 37°C incubator with 8% CO_2_ on an orbital shaker platform rotating at 125 rpm. Secreted fusion constructs were captured from the conditioned medium using protein A affinity chromatography. Bound proteins were eluted with 100 mM citrate buffer (pH 3.0) and immediately neutralized with 2 M Tris (pH 8.6). The eluate was dialyzed into phosphate‐buffered saline (PBS) (pH 7.5) and stored at −20 or −80°C. To generate soluble Cripto‐1, the Fc fusion protein was cleaved using TEV protease. The cleaved Fc fragment was removed by passing the sample over a protein A affinity column, and the flow‐through containing soluble Cripto‐1 was collected. Cleaved Cripto‐1 was further purified by SEC in PBS buffer.

### 5.4 Antibody generation

Custom monoclonal antibodies against cleaved, purified Cripto‐1 were generated by ABclonal. Mouse hybridoma supernatants were initially screened by ABclonal, followed by further analysis via SPR to identify those producing high‐affinity antibodies against distinct Cripto‐1 domains. Selected hybridomas were adapted for growth in serum‐free suspension media (CD Hybridoma, Life Technologies). Subsequently, antibodies were purified using Protein G affinity chromatography.

### 5.5 AlphaFold3 modeling

Human sequences for the Nodal growth factor region (residues 238–347), ALK4 ligand‐binding domain (residues 24–126), ACVR2B ligand‐binding domain (residues 19–137), and processed Cripto‐1 (residues 31–169), as well as their corresponding zebrafish homologs, were submitted simultaneously to the AlphaFold3 server to enable structural comparison of complexes across species (Abramson et al., 2024). Models were generated using three recycles, a tolerance of 0.1, and default settings for templates and extra MSAs. Model quality was assessed using predicted local distance difference test (pLDDT) and PAE scores. Predicted structures were visualized and figures were generated using PyMOL (version 2.5.2) (Schrodinger 2015).

### 5.6 Surface plasmon resonance

SPR experiments were performed using a Biacore 3000 at 25°C. Running buffer consisted of HBS/EPS (0.01 M Hepes, 0.5 M NaCl, 3 mM EDTA, 0.005% (v/v) Tween 20, pH 7.4) containing 0.1% bovine serum albumin. The experimental flow rate was 50 μL/min. Approximately 5000–7000 Response Units (RU) of either anti‐mouse IgG (Mouse antibody capture kit, Cytiva) or anti‐human IgG (Fc) (Human antibody capture kit, Cytiva) were immobilized on three channels of a CM5 chip using amine‐coupling chemistry. For hybridoma evaluation, 100–500 μL of CM were loaded on the anti‐mouse IgG chip's experimental flow channels. For binding analyses, approximately 500 RU of purified Cripto‐1 constructs, or 500 RU of ALK4‐Fc were loaded on the anti‐human IgG chip's experimental flow channels. A reference channel was used to account for nonspecific binding, drift, and bulk shifts.

To investigate the binding of Nodal to Cripto‐1 domains (N, E, C, NE, EC), 160 nM Nodal was injected over the different domain Cripto‐1 constructs. To identify binding epitopes of antibodies (G1H2, H11F4, H3A6), 160 nM of each purified mAb was injected over the different domain Cripto‐1 constructs. Single‐concentration experiments were used to obtain an estimate of binding parameters, and all experiments were repeated at least twice. To confirm kinetic parameters, titrations were carried out over several concentrations. To identify the Cripto‐1 domain binding to ALK4‐Fc, full‐length Cripto‐1 was pre‐incubated with varying concentrations of the different antibodies for 1 h at room temperature and then loaded on the experimental flow channel with immobilized ALK4‐Fc. Antibody‐crosslinked surfaces were regenerated to baseline after each binding cycle by injecting MgCl_2_. Sensorgrams were double‐referenced, and the processed data were fitted to a “1:1” or “bivalent analyte” binding model using BiaEvaluation software. The equilibrium‐binding constant (*K*_D_) was determined by calculating the ratio of binding rate constants (*k*_d_/*k*_a_). Results are summarized in Table 1.

### 5.7 Size exclusion chromatography

SEC was performed to analyze the oligomeric state of Cripto‐1‐Fc constructs alone and in complex with ALK4‐Fc. Briefly, different Cripto‐1‐Fc constructs (500 μg in 500 μL (12 μM final protein concentration)) were either loaded alone or preincubated with equimolar amounts of ALK4‐Fc μL (24 μM final protein concentration) for 30 min at room temperature to allow complex formation. The samples were then injected into a Superdex 200 10/300 GL column (Cytiva) pre‐equilibrated with PBS. The column was run at a flow rate of 0.5 mL/min, and 0.5 mL fractions were collected. The elution profiles were monitored by absorbance at 280 nm. Fractions corresponding to the elution volumes of Cripto‐1‐Fc constructs and/or ALK4‐Fc were analyzed by SDS‐PAGE under reducing conditions to confirm the presence and identity of the respective proteins. As a control, ALK4‐Fc alone (12 μM) was also run on the column under identical conditions to determine its elution volume.

### 5.8 Immunoblots

Approximately 5 × 10^5^ NTERA‐2 cl.D1 (NT2/D1) cells were plated per well in 6‐well plates and grown to confluence in DMEM modified to contain 2 mM L‐glutamine, 4500 mg/L glucose and 10% fetal bovine serum. For ligand signaling assays, cells were treated with 0.2 nM Activin A, Activin B, BMP‐4, BMP‐7, or TGF‐β1, or 4 nM Nodal for 30 min. For Cripto‐1 knockdown assays, approximately 1 × 10^5^ NT2/D1 cells in complete medium were seeded per well in 24‐well plates and grown overnight. Cells were transfected with 0.75 μL of Lipofectamine 3000, 500 ng of TDGF1 shRNA (Sigma, SHCLNG‐NM‐003212, clone ID 4890 and 4893) or scrambled shRNA control vector. After 48 h, cells were treated with 4 nM Nodal for 30 min. For antibody inhibition assays, 5 × 10^5^ NTERA‐2 cl.D1 (NT2/D1) cells were plated per well in 6‐well plates and induced with 5 ng/mL cells were pretreated with 300 nM anti‐Cripto‐1 antibodies for 30 min and induced with Activin A or 4 nM Nodal for another 30 min. To prepare samples for Western blot, protein lysates were prepared after 30 min of treatment using ice‐cold RIPA lysis buffer containing 1X “Protease Arrest” and 2X “Phosphatase Arrest” (G‐Biosciences) and stored at −80°C. Total protein concentration was determined using the BCA assay. For Western blot analysis, equal amounts of protein (typically 10 μg) were separated under reducing conditions on 12% TGX‐polyacrylamide gels (Bio‐Rad) and transferred to Hybond‐P membrane (GE Healthcare). Membranes were blocked with Superblock (Thermo Fisher) and incubated with primary antibodies from Cell Signaling at a 1:1000 dilution (e.g., anti‐phospho‐SMAD2/3 (138D4), anti‐phospho‐SMAD1/5/8 (41D10)) or 1:5000 dilution (e.g., anti‐*β*‐actin (8H10D10)). This was followed by incubation with horseradish peroxidase‐conjugated secondary antibody at 1:10,000 (Actin) or 1:2000 (SMADs) dilutions (7074). Western Bright ECL HRP substrate (Advansta) was used for detection, and blots were visualized using autoradiography film.

## AUTHOR CONTRIBUTIONS

**Kit‐Yee Chu:** Investigation; validation; writing – review and editing; formal analysis. **Amberly N. Crawford:** Investigation; visualization; writing – review and editing; formal analysis; validation. **Bradon S. Krah:** Investigation; formal analysis. **Vijayalakshmi Thamilselvan:** Investigation; validation; methodology; formal analysis. **Anjali Malik:** Investigation; methodology. **Nina A. Aitas:** Formal analysis. **Erik Martinez‐Hackert:** Conceptualization; funding acquisition; writing – original draft; validation; visualization; writing – review and editing; formal analysis; supervision; data curation; resources; project administration.

## FUNDING INFORMATION

This work was supported by NIH grant R01 GM121499 (to E.M.H.).

## CONFLICT OF INTEREST STATEMENT

E.M.H. is a founder of and holds equity in Advertent Biotherapeutics. K.‐Y.C., A.N.C., B.S.K., A.M, A.A.A., and V.T. declare no competing interests.

## Supporting information


**Figure S1:** AlphaFold3‐predicted structural model quality metrics for the human Cripto‐1–Nodal–ALK4–ACVR2B signaling complex.
**Figure S2:** Superposition of AlphaFold3‐predicted structural models and interaction analysis using FoldScript.
**Figure S3:** SPR sensorgrams of Nodal binding to various Cripto‐1 constructs.
**Figure S4:** SPR sensorgrams for hybridoma selection and antibody characterization.
**Figures S5–S7:** SPR sensorgrams of domain‐specific anti‐Cripto‐1 monoclonal antibodies binding to various Cripto‐1 constructs.
**Figure S8:** ALK4 sequence alignment and interaction analysis.
**Figure S9:** Western blots of nodal‐induced SMAD2/3 signaling in NTERA‐2 cells.
**Figure S10:** TGF‐*β* family ligand sequence alignment and interaction analysis.

## Data Availability

The data and materials that support the findings of this study are available from the corresponding author upon reasonable request. The processed data and statistical analyses are available in the manuscript and supporting information files. Raw data will be made available upon request. The atomic coordinates for the AlphaFold3‐predicted models of Cripto‐1–Nodal–ALK4–ACVR2B complexes are available from the corresponding author upon request. Antibodies are available upon request.

## References

[pro70034-bib-0001] Abramson J , Adler J , Dunger J , Evans R , Green T , Pritzel A , et al. Accurate structure prediction of biomolecular interactions with AlphaFold 3. Nature. 2024;630(8016):493–500. 38718835 10.1038/s41586-024-07487-w PMC11168924

[pro70034-bib-0002] Adachi H , Saijoh Y , Mochida K , Ohishi S , Hashiguchi H , Hirao A , et al. Determination of left/right asymmetric expression of nodal by a left side‐specific enhancer with sequence similarity to a lefty‐2 enhancer. Genes Dev. 1999;13(12):1589–1600. 10385627 10.1101/gad.13.12.1589 PMC316797

[pro70034-bib-0003] Adkins HB , Bianco C , Schiffer SG , Rayhorn P , Zafari M , Cheung AE , et al. Antibody blockade of the Cripto CFC domain suppresses tumor cell growth in vivo. J Clin Invest. 2003;112(4):575–587. 12925698 10.1172/JCI17788 PMC171388

[pro70034-bib-0004] Ang SL , Constam DB . A gene network establishing polarity in the early mouse embryo. Semin Cell Dev Biol. 2004;15(5):555–561. 15271301 10.1016/j.semcdb.2004.04.009

[pro70034-bib-0005] Aykul S , Ni W , Mutatu W , Martinez‐Hackert E . Human cerberus prevents nodal‐receptor binding, inhibits nodal signaling, and suppresses nodal‐mediated phenotypes. PLoS One. 2015;10(1):e0114954. 25603319 10.1371/journal.pone.0114954 PMC4300205

[pro70034-bib-0006] Aykul S , Parenti A , Chu KY , Reske J , Floer M , Ralston A , et al. Biochemical and cellular analysis reveals ligand binding specificities, a molecular basis for ligand recognition, and membrane association‐dependent activities of Cripto‐1 and cryptic. J Biol Chem. 2017;292(10):4138–4151. 28126904 10.1074/jbc.M116.747501 PMC5354514

[pro70034-bib-0007] Bell CH , Healey E , van Erp S , Bishop B , Tang C , Gilbert RJC , et al. Structure of the repulsive guidance molecule (RGM)‐neogenin signaling hub. Science. 2013;341(6141):77–80. 23744777 10.1126/science.1232322 PMC4730555

[pro70034-bib-0008] Bianco C , Adkins HB , Wechselberger C , Seno M , Normanno N , De Luca A , et al. Cripto‐1 activates nodal‐ and alk4‐dependent and ‐independent signaling pathways in mammary epithelial cells. Mol Cell Biol. 2002;22(8):2586–2597. 11909953 10.1128/MCB.22.8.2586-2597.2002 PMC133714

[pro70034-bib-0009] Blanchet MH , Le Good JA , Oorschot V , Baflast S , Minchiotti G , Klumperman J , et al. Cripto localizes nodal at the limiting membrane of early endosomes. Sci Signal. 2008;1(45):ra13. 19001664 10.1126/scisignal.1165027

[pro70034-bib-0010] Calvanese L , Saporito A , Marasco D , D'Auria G , Minchiotti G , Pedone C , et al. Solution structure of mouse Cripto CFC domain and its inactive variant Trp107Ala. J Med Chem. 2006;49(24):7054–7062. 17125258 10.1021/jm060772r

[pro70034-bib-0011] Castonguay R , Werner ED , Matthews RG , Presman E , Mulivor AW , Solban N , et al. Soluble endoglin specifically binds bone morphogenetic proteins 9 and 10 via its orphan domain, inhibits blood vessel formation, and suppresses tumor growth. J Biol Chem. 2011;286(34):30034–30046. 21737454 10.1074/jbc.M111.260133 PMC3191044

[pro70034-bib-0012] Dono R , Montuori N , Rocchi M , De Ponti‐Zilli L , Ciccodicola A , Persico MG . Isolation and characterization of the Cripto autosomal gene and its x‐linked related sequence. Am J Hum Genet. 1991;49(3):555–565. 1882841 PMC1683146

[pro70034-bib-0013] Gray PC , Harrison CA , Vale W . Cripto forms a complex with activin and type ii activin receptors and can block activin signaling. Proc Natl Acad Sci USA. 2003;100(9):5193–5198. 12682303 10.1073/pnas.0531290100 PMC154321

[pro70034-bib-0014] Gray PC , Shani G , Aung K , Kelber J , Vale W . Cripto binds transforming growth factor beta (TGF‐beta) and inhibits TGF‐beta signaling. Mol Cell Biol. 2006;26(24):9268–9278. 17030617 10.1128/MCB.01168-06 PMC1698529

[pro70034-bib-0015] Gritsman K , Zhang J , Cheng S , Heckscher E , Talbot WS , Schier AF . The EGF‐CFC protein one‐eyed pinhead is essential for nodal signaling. Cell. 1999;97(1):121–132. 10199408 10.1016/s0092-8674(00)80720-5

[pro70034-bib-0016] Gu Z , Nomura M , Simpson BB , Lei H , Feijen A , van den Eijnden‐van RJ , et al. The type i activin receptor ActRIB is required for egg cylinder organization and gastrulation in the mouse. Genes Dev. 1998;12(6):844–857. 9512518 10.1101/gad.12.6.844 PMC316628

[pro70034-bib-0017] Guardiola O , Iavarone F , Nicoletti C , Ventre M , Rodriguez C , Pisapia L , et al. Cripto‐based micro‐heterogeneity of mouse muscle satellite cells enables adaptive response to regenerative microenvironment. Dev Cell. 2023;58(24):2896–2913 e2896. 38056454 10.1016/j.devcel.2023.11.009 PMC10855569

[pro70034-bib-0018] Guardiola O , Lafuste P , Brunelli S , Iaconis S , Touvier T , Mourikis P , et al. Cripto regulates skeletal muscle regeneration and modulates satellite cell determination by antagonizing myostatin. Proc Natl Acad Sci USA. 2012;109(47):E3231–E3240. 23129614 10.1073/pnas.1204017109 PMC3511144

[pro70034-bib-0019] Healey EG , Bishop B , Elegheert J , Bell CH , Padilla‐Parra S , Siebold C . Repulsive guidance molecule is a structural bridge between neogenin and bone morphogenetic protein. Nat Struct Mol Biol. 2015;22(6):458–465. 25938661 10.1038/nsmb.3016 PMC4456160

[pro70034-bib-0020] Heldin CH , Moustakas A . Signaling receptors for TGF‐beta family members. Cold Spring Harb Perspect Biol. 2016;8(8):1–35. 10.1101/cshperspect.a022053 PMC4968163 27481709

[pro70034-bib-0021] Hinck AP , Mueller TD , Springer TA . Structural biology and evolution of the TGF‐beta family. Cold Spring Harb Perspect Biol. 2016;8(12):1–53. 10.1101/cshperspect.a022103 PMC5131774 27638177

[pro70034-bib-0022] Kelber JA , Shani G , Booker EC , Vale WW , Gray PC . Cripto is a noncompetitive activin antagonist that forms analogous signaling complexes with activin and nodal. J Biol Chem. 2008;283(8):4490–4500. 18089557 10.1074/jbc.M704960200

[pro70034-bib-0023] Kim SK , Henen MA , Hinck AP . Structural biology of betaglycan and endoglin, membrane‐bound co‐receptors of the TGF‐beta family. Exp Biol Med. 2019;244(17):1547–1558. 10.1177/1535370219881160 PMC6920675 31601110

[pro70034-bib-0024] Kirsch T , Sebald W , Dreyer MK . Crystal structure of the BMP‐2‐BRIA ectodomain complex. Nat Struct Biol. 2000;7(6):492–496. 10881198 10.1038/75903

[pro70034-bib-0025] Liguori GL , Borges AC , D'Andrea D , Liguoro A , Goncalves L , Salgueiro AM , et al. Cripto‐independent nodal signaling promotes positioning of the A‐P axis in the early mouse embryo. Dev Biol. 2008;315(2):280–289. 18241853 10.1016/j.ydbio.2007.12.027

[pro70034-bib-0026] Perrimon N , Pitsouli C , Shilo BZ . Signaling mechanisms controlling cell fate and embryonic patterning. Cold Spring Harb Perspect Biol. 2012;4(8):a005975. 22855721 10.1101/cshperspect.a005975 PMC3405863

[pro70034-bib-0027] Prezioso C , Iaconis S , Andolfi G , Zentilin L , Iavarone F , Guardiola O , et al. Conditional Cripto overexpression in satellite cells promotes myogenic commitment and enhances early regeneration. Front Cell Dev Biol. 2015;3:31. 26052513 10.3389/fcell.2015.00031 PMC4439575

[pro70034-bib-0028] Reissmann E , Jornvall H , Blokzijl A , Andersson O , Chang C , Minchiotti G , et al. The orphan receptor ALK7 and the activin receptor ALK4 mediate signaling by nodal proteins during vertebrate development. Genes Dev. 2001;15(15):2010–2022. 11485994 10.1101/gad.201801 PMC312747

[pro70034-bib-0029] Saito T , Bokhove M , Croci R , Zamora‐Caballero S , Han L , Letarte M , et al. Structural basis of the human endoglin‐BMP9 interaction: insights into BMP signaling and HHT1. Cell Rep. 2017;19(9):1917–1928. 28564608 10.1016/j.celrep.2017.05.011 PMC5464963

[pro70034-bib-0030] Schier AF . Nodal signaling in vertebrate development. Annu Rev Cell Dev Biol. 2003;19:589–621. 14570583 10.1146/annurev.cellbio.19.041603.094522

[pro70034-bib-0031] Schiffer SG , Foley S , Kaffashan A , Hronowski X , Zichittella AE , Yeo CY , et al. Fucosylation of cripto is required for its ability to facilitate nodal signaling. J Biol Chem. 2001;276(41):37769–37778. 11500501 10.1074/jbc.M104774200

[pro70034-bib-0032] Schrodinger, LLC . The pymol molecular graphics system, version 1.8. Forthcoming, 2015.

[pro70034-bib-0033] Shi S , Ge C , Luo Y , Hou X , Haltiwanger RS , Stanley P . The threonine that carries fucose, but not fucose, is required for Cripto to facilitate nodal signaling. J Biol Chem. 2007;282(28):20133–20141. 17504756 10.1074/jbc.M702593200

[pro70034-bib-0034] Shi Y , Massague J . Mechanisms of TGF‐beta signaling from cell membrane to the nucleus. Cell. 2003;113(6):685–700. 12809600 10.1016/s0092-8674(03)00432-x

[pro70034-bib-0035] Sonnen KF , Janda CY . Signalling dynamics in embryonic development. Biochem J. 2021;478(23):4045–4070. 34871368 10.1042/BCJ20210043 PMC8718268

[pro70034-bib-0036] Townson SA , Martinez‐Hackert E , Greppi C , Lowden P , Sako D , Liu J , et al. Specificity and structure of a high affinity activin receptor‐like kinase 1 (ALK1) signaling complex. J Biol Chem. 2012;287(33):27313–27325. 22718755 10.1074/jbc.M112.377960 PMC3431715

[pro70034-bib-0037] Watanabe K , Meyer MJ , Strizzi L , Lee JM , Gonzales M , Bianco C , et al. Cripto‐1 is a cell surface marker for a tumorigenic, undifferentiated subpopulation in human embryonal carcinoma cells. Stem Cells. 2010;28(8):1303–1314. 20549704 10.1002/stem.463 PMC3069615

[pro70034-bib-0038] Wouters MA , Rigoutsos I , Chu CK , Feng LL , Sparrow DB , Dunwoodie SL . Evolution of distinct EGF domains with specific functions. Protein Sci. 2005;14(4):1091–1103. 15772310 10.1110/ps.041207005 PMC2253431

[pro70034-bib-0039] Wu MY , Hill CS . TGF‐beta superfamily signaling in embryonic development and homeostasis. Dev Cell. 2009;16(3):329–343. 19289080 10.1016/j.devcel.2009.02.012

[pro70034-bib-0040] Yan YT , Gritsman K , Ding J , Burdine RD , Corrales JD , Price SM , et al. Conserved requirement for EGF‐CFC genes in vertebrate left‐right axis formation. Genes Dev. 1999;13(19):2527–2537. 10521397 10.1101/gad.13.19.2527 PMC317064

[pro70034-bib-0041] Yan YT , Liu JJ , Luo Y , Chaosu E , Haltiwanger RS , Abate‐Shen C , et al. Dual roles of Cripto as a ligand and coreceptor in the nodal signaling pathway. Mol Cell Biol. 2002;22(13):4439–4449. 12052855 10.1128/MCB.22.13.4439-4449.2002 PMC133918

[pro70034-bib-0042] Yeo C , Whitman M . Nodal signals to smads through Cripto‐dependent and Cripto‐independent mechanisms. Mol Cell. 2001;7(5):949–957. 11389842 10.1016/s1097-2765(01)00249-0

[pro70034-bib-0043] Zinski J , Tajer B , Mullins MC . TGF‐beta family signaling in early vertebrate development. Cold Spring Harb Perspect Biol. 2018;10(6):1–77. 10.1101/cshperspect.a033274 PMC5983195 28600394

