# Supplementary material for: Cripto‐1 acts as a molecular bridge linking nodal to ALK4 via distinct structural domains
Source: Protein Sci. 2025 Jan 22;34(2):e70034. doi: 10.1002/pro.70034 (PMC11751877; doi:10.1002/pro.70034)
Supplement: Supplementary file 1 — Figure S1: AlphaFold3‐predicted structural model quality metrics for the human Cripto‐1–Nodal–ALK4–ACVR2B signaling complex. Figure S2: Superposition of AlphaFold3‐predicted structural models and interaction analysis using FoldScript. Figure S3: SPR sensorgrams of Nodal binding to various Cripto‐1 constructs. Figure S4: SPR sensorgrams for hybridoma selection and antibody characterization. Figures S5–S7: SPR sensorgrams of domain‐specific anti‐Cripto‐1 monoclonal antibodies binding to various Cripto‐1 constructs. Figure S8: ALK4 sequence alignment and interaction analysis. Figure S9: Western blots of nodal‐induced SMAD2/3 signaling in NTERA‐2 cells. Figure S10: TGF‐β family ligand sequence alignment and interaction analysis. [file PRO-34-e70034-s001.pdf]

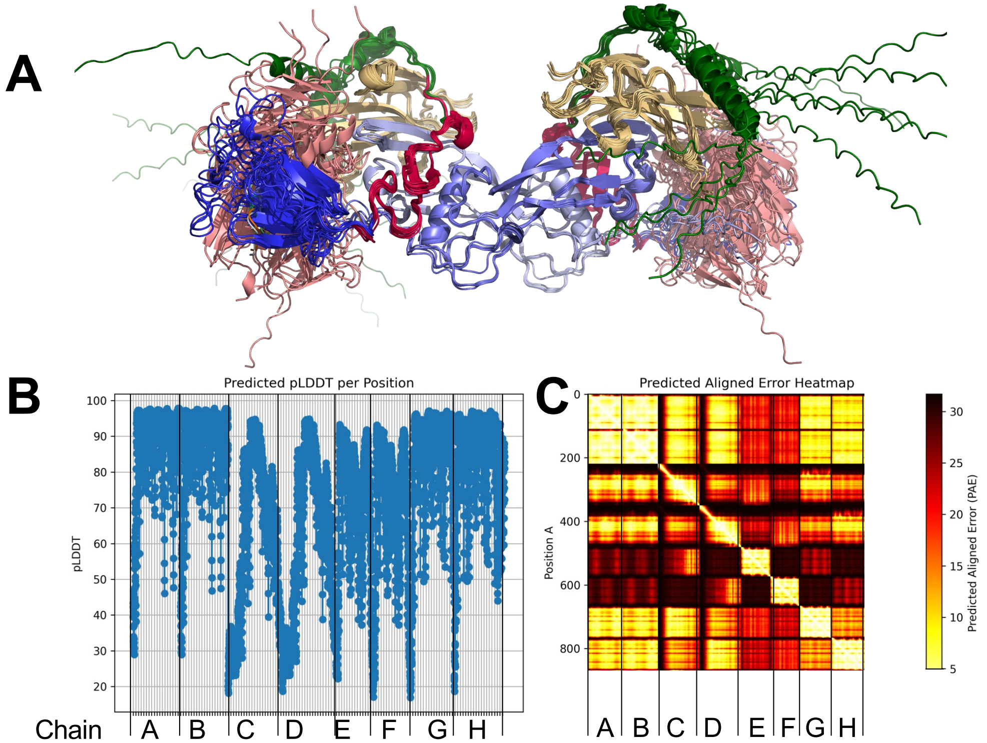

Figure S1: AlphaFold3-predicted structural models of the human and zebrafish Cripto-1-Nodal-ALK4-ACVR2B signaling complex. (A) Superposition of the top 5 human and top 5 zebrafish Nodal-Cripto-1-ALK4-ACVR2B models based on Nodal protomer A. Nodal is shown in light blue, ALK4 in salmon, ACVR2B in gold, and Cripto-1 is colored by domain: green (N-domain), red (EGF domain), and blue (CFC domain). The superposition reveals movement in the hinge region connecting the Cripto-1 EGF and CFC domains, resulting in apparent structural variability that reflects the inherent flexibility of Cripto-1. (B) Predicted Local Distance Difference Test (pLDDT) per position, indicating the confidence in the predicted structure.(C) Aligned Error (PAE) for the top-ranked human Nodal-Cripto-1-ALK4-ACVR2B model, reflecting the expected error in the relative positions of aligned residues. The sequence coverage is as follows: chains A and B correspond to human Nodal, chains C and D to human Cripto-1, chains E and F to human ALK4, and chains G and H to human ACVR2B.

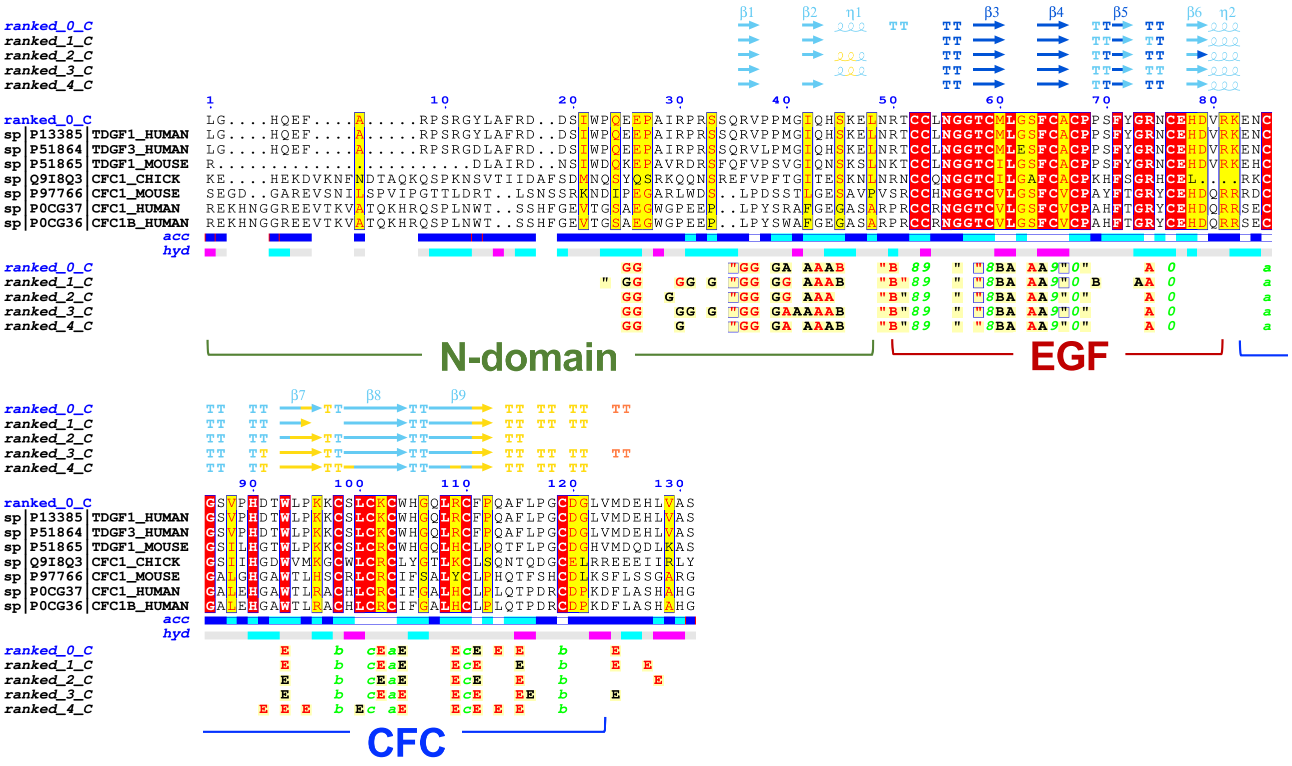

Figure S2: Sequence Alignment and Interaction Analysis. This figure shows a sequence alignment of Cripto-1 homologs, including human Cripto-1 (TDGF1_Human). Below the alignment, colored letters indicate residues in human Cripto-1 predicted to interact with specific chains in the AlphaFold3-predicted Cripto-1/Nodal/ALK4/ACVR2B complex. Each line of letters corresponds to a different AlphaFold3 rank model (as indicated on the side of the alignment). Each letter represents the chain contacted by the Cripto-1 residue above it. (A/B = Nodal chains, E = ALK4, G = ACVR2B). Red letter: Contact distance < 3.2 Å. Black letter: Contact distance between 3.2 Å and 3.7 Å. Interaction analysis was performed using FoldScript and human Cripto-1 models.

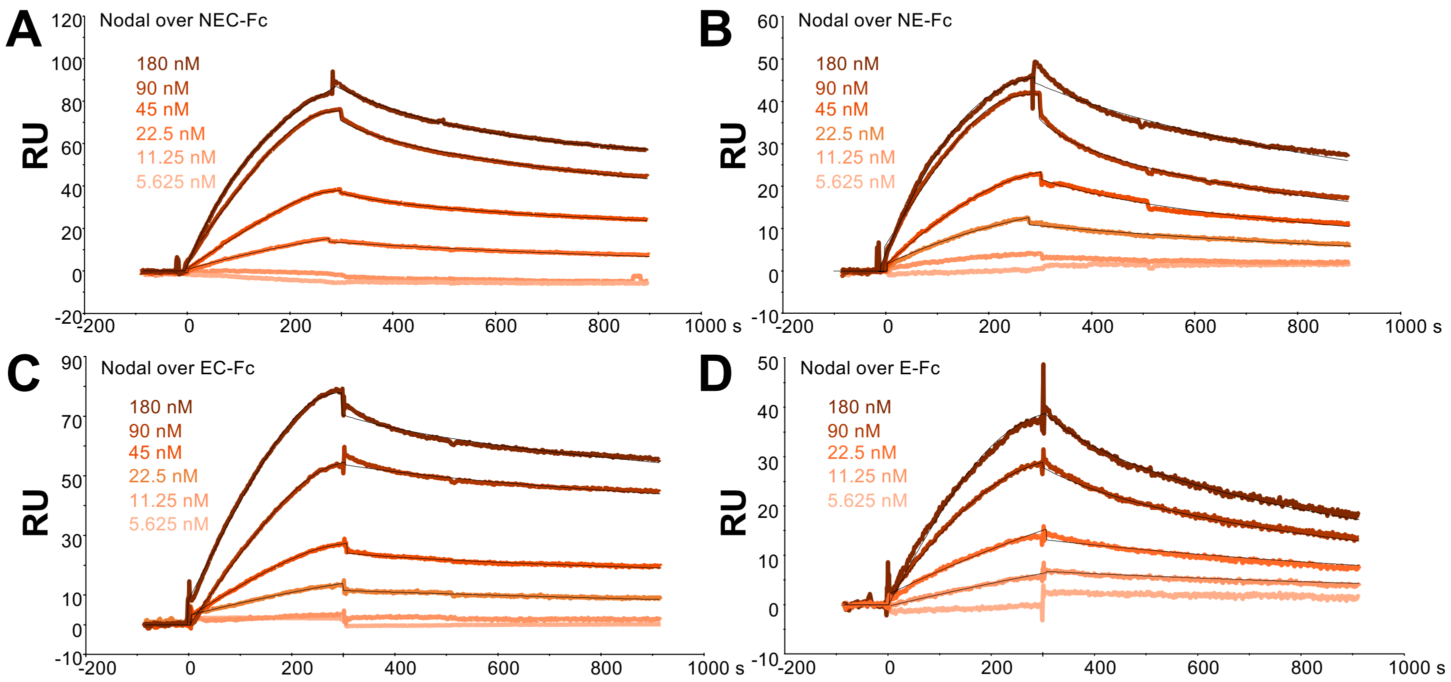

Figure S3: SPR sensorgrams depicting the binding of increasing concentrations of Nodal to various immobilized Cripto-1 constructs. (A) NEC-Fc, (B) NE-Fc, (C) EC-Fc, and (D) E-Fc. Nodal did not bind to N-Fc or C-Fc (not shown). Curves are color-coded by Nodal concentration (nM), and modeled curves are shown as black lines.

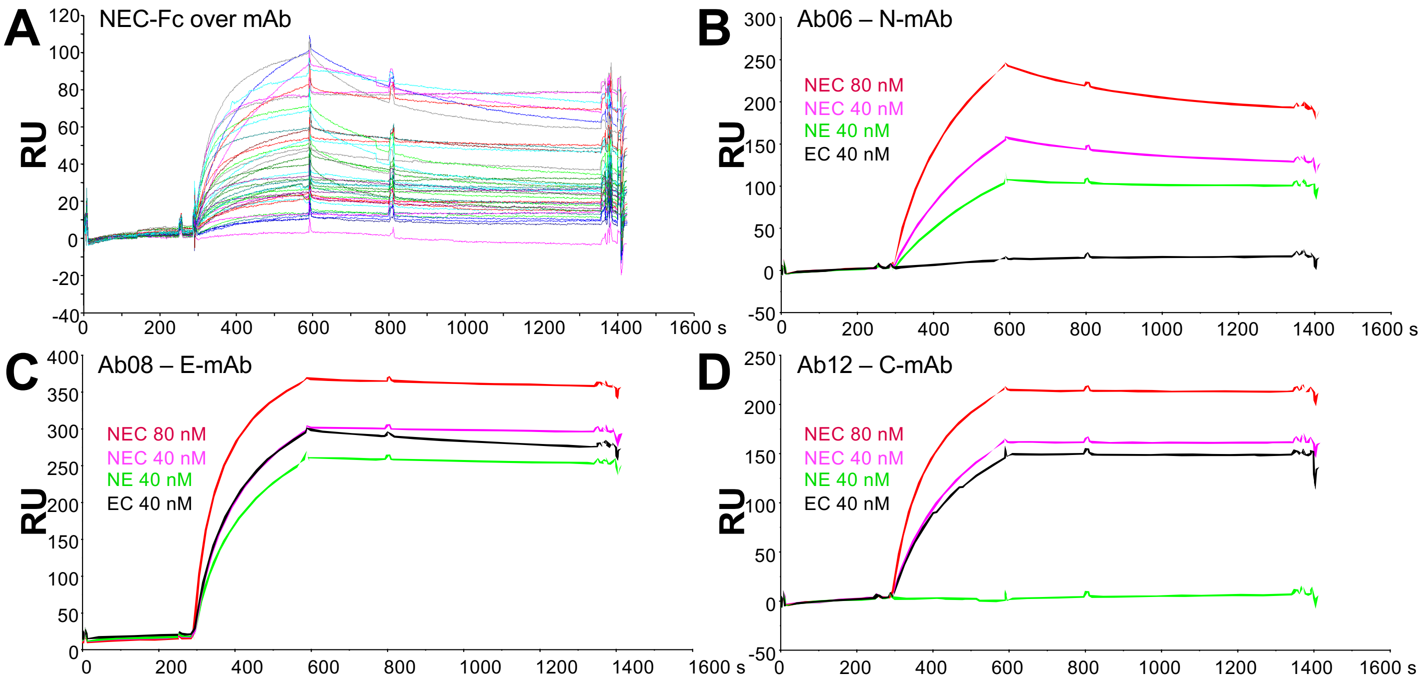

Figure S4: SPR sensorgrams depicting hybridoma selection and preliminary antibody characterization from hybridoma conditioned medium (CM). Antibodies were captured from hybridoma CM using an anti-mouse antibody capture kit, and full-length Cripto-1-Fc or Cripto-1-Fc deletion constructs were injected. (A) Preliminary antibody selection. 500 µL of ELISA-positive hybridoma clone CM were loaded onto an SPR sensor chip crosslinked with anti-mouse Fc antibody. Full-length Cripto-1-Fc (150 µL of 80 nM NEC-Fc) was injected over the captured antibodies. Association and dissociation curves provide an indication of the respective antibody binding affinity, and the response magnitude (RU) provides an indication of hybridoma productivity. (B-D) Preliminary epitope mapping. Representative examples of antibodies picked from our original CM capture experiments. 100 µL hybridoma CM was injected over an SPR sensor chip crosslinked with anti-mouse Fc antibody. 80 nM (red) or 40 nM (magenta) NEC-Fc, or 40 nM NE-Fc (green) or 40 nM EC-Fc (black) were injected over the captured antibodies. (B) Ab06 is an N-domain-specific mAb, as it binds NEC-Fc and NE-Fc, but not EC-Fc. (C) Ab08 is an EGF-like-domain-specific mAb, as it binds all three Cripto-1 constructs. (D) Ab03 is a C-domain-specific mAb, as it binds NEC-Fc and EC-Fc, but not NE-Fc

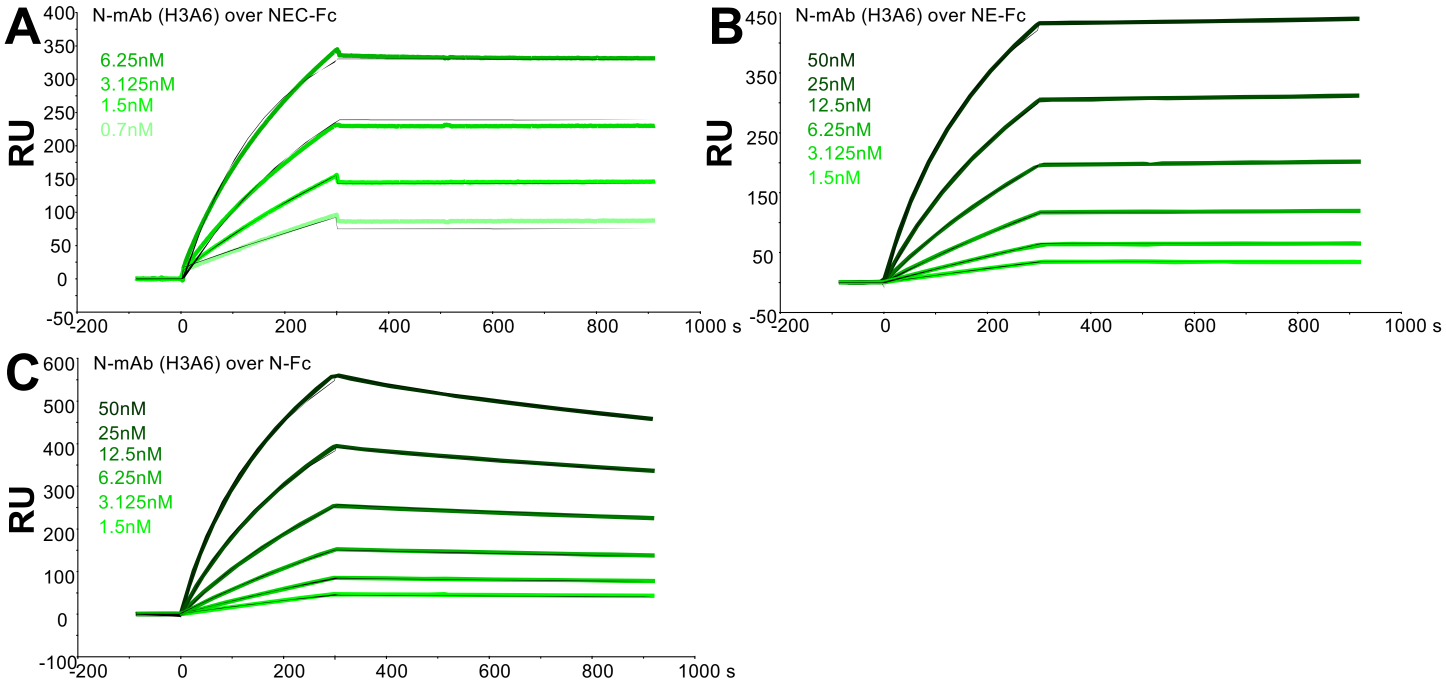

Figure S5: SPR sensorgrams depicting the binding of increasing concentrations of N-mAb to various immobilized Cripto-1 constructs. (A) NEC-Fc, (B) NE-Fc, and (C) N-Fc. The N-mAb did not bind to EC-Fc, E-Fc, or C-Fc (not shown). Curves are color-coded by N-mAb concentration (nM), and modeled curves are shown as black lines.

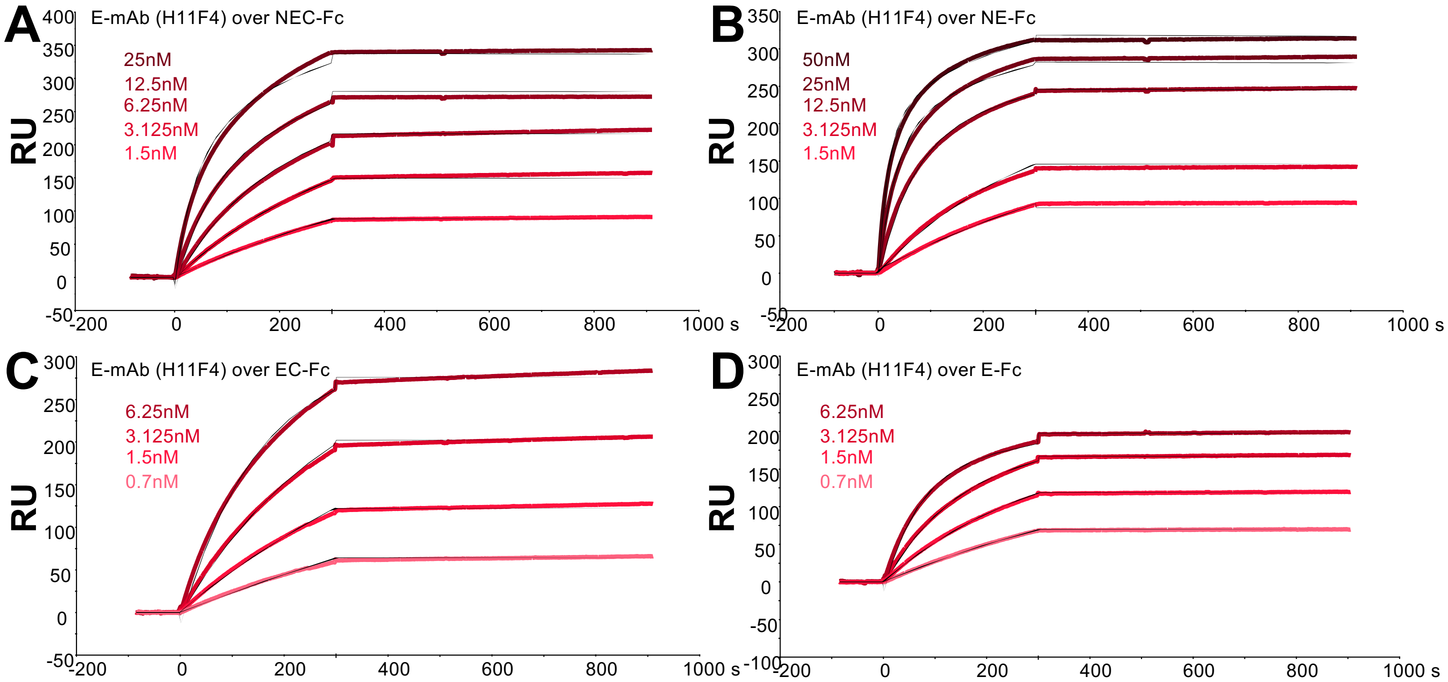

Figure S6: SPR sensorgrams depicting the binding of increasing concentrations of E-mAb to various immobilized Cripto-1 constructs. (A) NEC-Fc, (B) NE-Fc, (C) EC-Fc, and (D) E-Fc. The E-mAb did not bind to N-Fc, or C-Fc (not shown). Curves are color-coded by E-mAb concentration (nM), and modeled curves are shown as black lines.

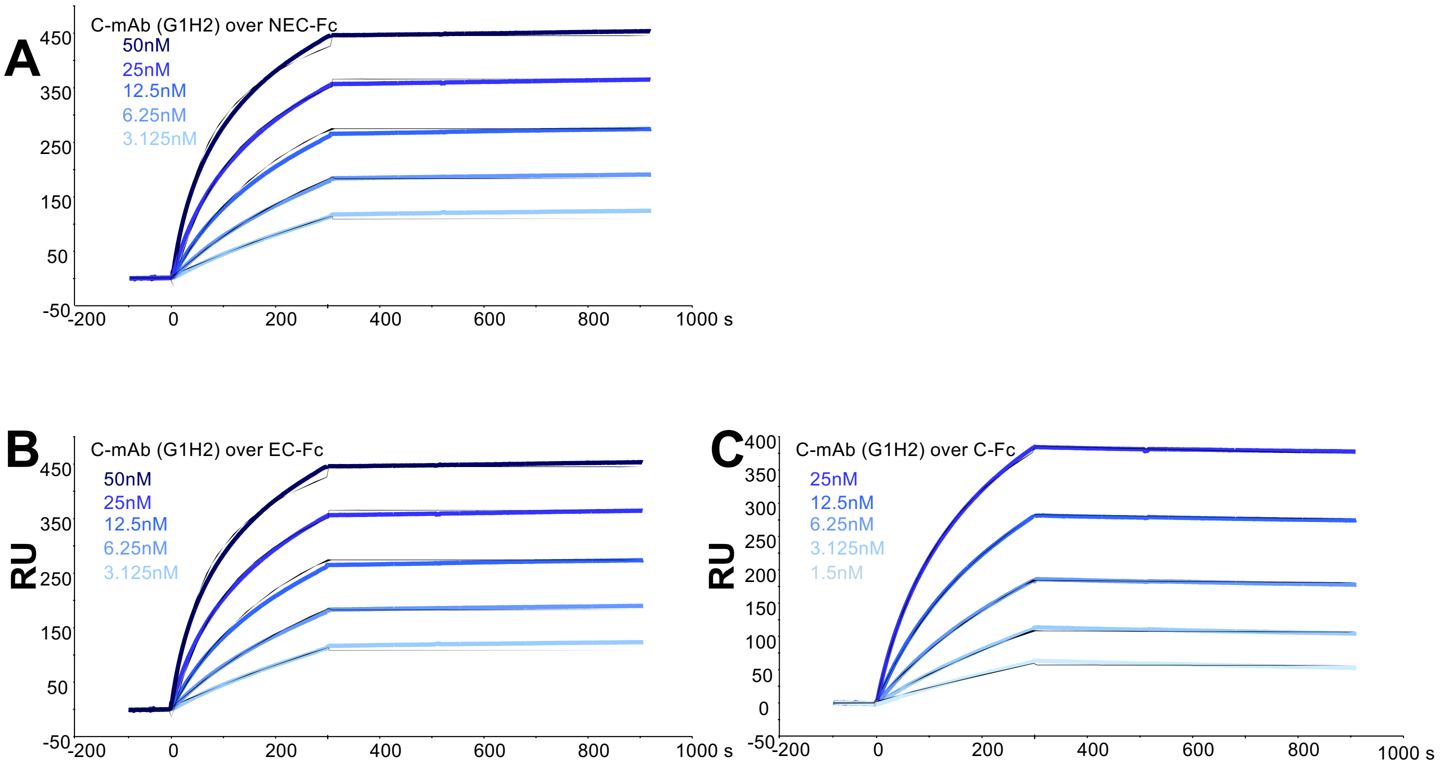

Figure S7: SPR sensorgrams depicting the binding of increasing concentrations of C-mAb to various immobilized Cripto-1 constructs. (A) NEC-Fc, EC-Fc, and (C) C-Fc. The C-mAb did not bind to NE-Fc, E-Fc, or N-Fc (not shown). Curves are color-coded by C-mAb concentration (nM), and modeled curves are shown as black lines.

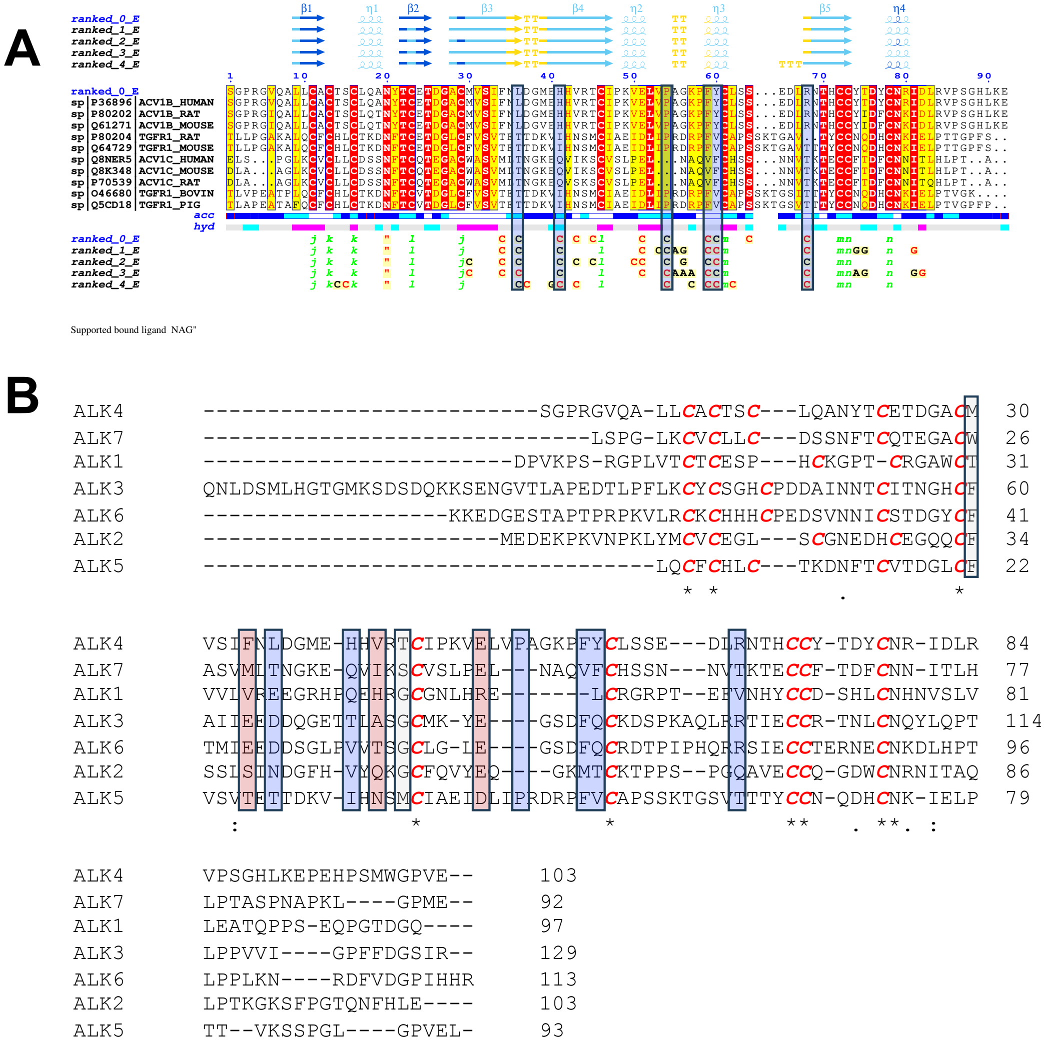

Figure S8: ALK4 Sequence Alignment and Interaction Analysis. **(A)** This figure shows a FoldScript alignment of ALK4 highlighting residues contacted by Cripto-1 predicted by AlphaFold3. ALK4 (ACVR1B) residues marked by the letter 'C' below the alignment are predicted to interact with Cripto-1 in the AlphaFold3-predicted complex. ALK4 residues identified as contacting Cripto-1 in all 5 AlphaFold3 models are highlighted by the blue bar. Each line of letters corresponds to a different AlphaFold3 rank model (as indicated on the side of the alignment). **(B)** The bottom panel shows a sequence alignment of all human type I receptors, including ALK4. The blue boxes identify residues that contact Cripto-1 in all AlphaFold3 models, the red boxes indicate residues that contact Cripto-1 in 3 or 4 AlphaFold3 models, and the grey boxes indicate residues that contact Cripto-1 in 2 AlphaFold3 models. Interaction analysis was performed using FoldScript.

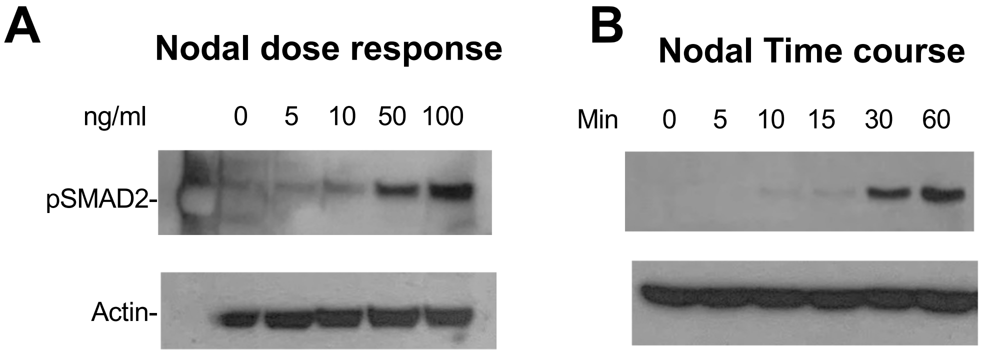

Figure S9: Nodal-induced SMAD2/3 signaling in NTERA-2 cells. (A) Western blot analysis of pSMAD2/3 levels in NTERA-2 cells treated with increasing concentrations of Nodal (0, 5, 10, 50, and 100 ng/mL) for 1 hour. (B) Western blot analysis of pSMAD2/3 levels in NTERA-2 cells treated with 100 ng/mL Nodal for various durations (0, 5, 10, 15, 30, and 60 minutes). β-actin serves as a loading control in both panels.

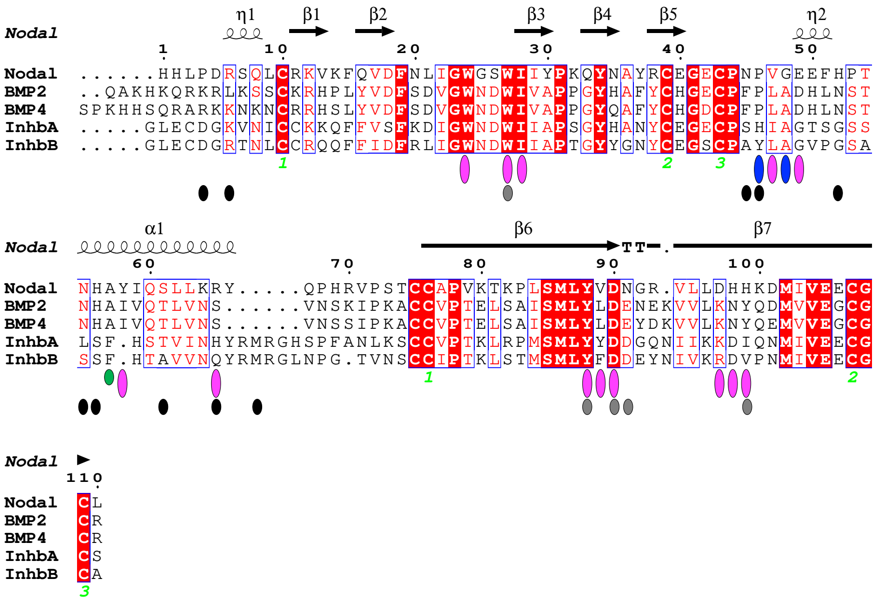

Figure S10: TGF-β Family Ligand Sequence Alignment and Interaction Analysis. This figure shows a ClustalW alignment of Nodal, BMP2, BMP4, activin A, and activin B displayed using ESPript. Residues identified using FoldScript analysis of the AlphaFold3 human Cripto-1–Nodal–ALK4–ACVR2B models that contact Cripto-1 are marked by ovals (purple shows Cripto-1 amino acid contacts, blue shows Cripto-1 T88Fucose contacts). The black and grey circles show activin A residues identified in the AlphaFold3 predicted activin A-ALK4 complex that are contacted by ALK4, where grey and black denote distinct activin B protomers. Nodal residues contacting the Cripto-1 fucose are partially conserved in BMPs, offering a rationale as to why BMP4 might bind Cripto-1. These sequences are significantly different in activins.
